# Systems Analysis Reveals Contraceptive-Induced Alteration of Cervicovaginal Gene Expression in a Randomized Trial

**DOI:** 10.3389/frph.2022.781687

**Published:** 2022-03-03

**Authors:** Christina Balle, Prachi M. Gupta, Gregory K. Tharp, Sydney A. Nelson, Iyaloo N. Konstantinus, Katie Lennard, Shameem Z. Jaumdally, Anna-Ursula Happel, Shaun L. Barnabas, Katherine Gill, Linda-Gail Bekker, Jo-Ann S. Passmore, Heather B. Jaspan, Steven E. Bosinger

**Affiliations:** ^1^Department of Pathology, Institute of Infectious Disease and Molecular Medicine, University of Cape Town, Cape Town, South Africa; ^2^Yerkes Genomics Core Laboratory, Yerkes National Primate Research Center, Atlanta, GA, United States; ^3^Namibia Institute of Pathology, Windhoek, Namibia; ^4^Department of Integrative Biomedical Sciences, University of Cape Town, Cape Town, South Africa; ^5^Desmond Tutu Health Centre, University of Cape Town, Cape Town, South Africa; ^6^Family Clinical Research Center, Stellenbosch University, Tygerberg, South Africa; ^7^National Health Laboratory Service, Cape Town, South Africa; ^8^Center for Global Infectious Disease Research, Seattle Children's Research Institute, Seattle, WA, United States; ^9^Department of Pediatrics and Global Health, University of Washington, Seattle, WA, United States; ^10^Department of Pathology and Laboratory Medicine, Emory University School of Medicine, Atlanta, GA, United States; ^11^Emory Vaccine Center, Emory University, Atlanta, GA, United States

**Keywords:** adolescents, cervicovaginal environment, hormonal contraception, randomized trial, South Africa, HIV, systems biology

## Abstract

Hormonal contraceptives (HCs) are vital in managing the reproductive health of women. However, HC usage has been linked to perturbations in cervicovaginal immunity and increased risk of sexually transmitted infections. Here, we evaluated the impact of three HCs on the cervicovaginal environment using high-throughput transcriptomics. From 2015 to 2017, 130 adolescent females aged 15–19 years were enrolled into a substudy of UChoose, a single-site, open-label randomized, crossover trial (NCT02404038) and randomized to injectable norethisterone–enanthate (Net-En), combined oral contraceptives (COC), or etonorgesterol/ethinyl–estradiol–combined contraceptive vaginal ring (CCVR). Cervicovaginal samples were collected after 16 weeks of randomized HC use and analyzed by RNA-Seq, 16S rRNA gene sequencing, and Luminex analysis. Participants in the CCVR arm had a significant elevation of transcriptional networks driven by IL-6, IL-1, and NFKB, and lower expression of genes supporting epithelial barrier integrity. An integrated multivariate analysis demonstrated that networks of microbial dysbiosis and inflammation best discriminated the CCVR arm from the other contraceptive groups, while genes involved in epithelial cell differentiation were predictive of the Net-En and COC arms. Collectively, these data from a randomized trial represent the most comprehensive “omics” analyses of the cervicovaginal response to HCs and provide important mechanistic guidelines for the provision of HCs in sub-Saharan Africa.

## Introduction

Young women in sub-Saharan Africa are at a high risk of unintended pregnancies (1, 2), which are associated with maternal and infant mortality and morbidity, especially in developing countries (3). Hormonal contraceptives (HCs) play a crucial role in preventing unintended pregnancy. However, in recent years, several studies have indicated that the use of various HCs has diverse effects on the female reproductive tract (FRT) mucosal environment, including the recruitment of HIV target cells (4, 5), changes in microbiota (6–9), and inflammation (10–12), with the potential to increase the risk of sexually transmitted infections (STIs) including HIV (13–16).

Several recent studies have indicated that HCs, in particular regimens utilizing the intramuscular progestin-only injection depot medroxyprogesterone acetate (DMPA-IM), can alter the epithelial barrier function. Human DMPA-IM use has been associated with reduced levels of epithelial growth factors (17–21), decreased levels of epithelial repair, cell junction, and maintenance proteins (22–24), and reduced levels of protease inhibitors, including matrix metalloproteinase (MMPs) and MMP tissue inhibitors (TIMPs) (18, 22, 25, 26) in the FRT. Data from the CAPRISA004 trial found that women using the injectable norethisterone-enanthate (Net-En) had reduced levels of cervicovaginal growth factors compared to women who did not use any HCs, suggesting a similar effect to that of DMPA-IM (27). In nonhuman primates (NHPs), DMPA treatment has been associated with a decrease in the vaginal epithelial thickness (28–30), more so than estrogen-containing combined oral contraceptives (COCs) (30, 31). Physiological levels of medroxyprogesterone acetate (MPA) reduced epithelial barrier integrity in the genital tract epithelial cells *in vitro* (32–34), whereas estradiol treatment led to enhanced barrier function (33, 34). Similarly, rodents and NHPs treated with DMPA had reduced genital levels of the cell–cell adhesion molecules and weakened epithelial barrier function that could be reversed by treatment with exogenous estrogen (35, 36). However, vaginal epithelial thinning in humans using DMPA–IM has not been demonstrated at the dose administered (6, 37–39).

In recent work, we have characterized HC-related perturbations to the vaginal microbiota (9), STI incidence (40), distribution of endocervical CD4+ T lymphocyte, T-helper 17 (Th17) cells (5), and cervicovaginal Th17-related and proinflammatory cytokines (5, 9) in South African adolescents enrolled into a substudy of UChoose, an open-label randomized, crossover study, comparing acceptability and contraceptive product preference of three HCs, the long-acting progestin-only injectable Net-En, COCs (Triphasil^TM^ or Nordette^TM^) or NuvaRing^®^, a combined etonorgesterol/ethinyl estradiol contraceptive vaginal ring (CCVR) (41). We found that (i) adolescents randomized to Net-En and CCVR had high vaginal microbial diversity and abundance of HIV risk-associated taxa compared to COC (9), (ii) that CCVR use was associated with an increased risk of *Neissearia gonorrheae* relative to Net-En and COC (40), and that (iii) CCVR usage was associated with increased levels of proinflammatory cytokines (5, 9). Here, we evaluated the effects of Net-En, COC, and CCVR on the adolescent host FRT transcriptome in the UChoose cohort (41) by using bulk RNA-Seq of cervicovaginal cytobrush samples. Furthermore, we aimed to assess the relationship between the transcriptome and the cervicovaginal microbiota and cytokines in relation to HC use.

## Materials and Methods

### Study Design

Adolescents were recruited through a parent study, UChoose, a single-site, open-label, randomized crossover study designed to evaluate the feasibility of different HC options among adolescents (ClinicalTrials.gov NCT02404038) (41). The parent study was approved by the Division of AIDS and the University of Cape Town Health Science Research Ethics Committee (HREC 801/2014) and was conducted in full compliance with South African Good Clinical Practice (SA-GCP) and ICH76 GCP and ICMJE guidelines. Approval for this substudy was obtained from the Human Research Ethics Committee at the University of Cape Town (HREC 801/2014). Participants 18 years or older provided informed consent for the substudy, while assent from the participant and informed consent from a parent or legal guardian were obtained for participants younger than 18 years old. Eligibility criteria for enrolment have been described in detail previously (41). Furthermore, participants were asked to abstain from inserting any nonstudy products or objects into the vagina throughout the duration of the study. Enrolment was within 40 days of the screening visit. The parent study enrolled 130 HIV-negative, nonpregnant adolescent girls aged 15–19 years between September 22, 2015 and June 30, 2017, all of whom consented to participate in this substudy. At enrolment, participants were randomly assigned in a 1:1:1 ratio to one of three study arms for a 16-week period. Arm 1: injectable HC (Net-En; containing 200 mg of the progestogen norethisterone enantate) once every 8 weeks; arm 2: vaginal ring contraception (CCVR, NuvaRing^®^ MSD Pty Ltd, containing etonogestrel/ethinyl estradiol, 0.120 mg/0.015 mg per day) to be inserted once every 28 days (and removed after 21 days) or arm 3: oral contraceptives [COC, either Triphasil^TM^ or Nordette^TM^; both containing ethinyl estradiol/levonorgestrel (Triphasil^TM^ (triphasic regimen): six tablets containing 30 μg/50 μg, five tablets containing 40 μg/75 μg, ten tablets containing 30 μg/125 μg; Nordette^TM^ (monophasic regimen): all tablets contain 30 μg/150 μg) daily for 21 days each month with a placebo tablet for 7 days]. Randomization was performed using random number sequence in Stata and provided to the pharmacist in sealed envelopes.

### Sample Collection

At all study visits (baseline and 16 weeks after HC initiation), a rapid HIV and a pregnancy test, were performed and if positive, the participant was counseled and referred. A detailed interviewer-assisted questionnaire assessing the participant's medical history and sexual behavior was completed. The following genital tract samples were collected at the baseline visit and 16 weeks after HC initiation: two vulvo-vaginal swabs for STI testing, Nugent scoring, *Candida* screening and pH measurement, a lateral wall swab for 16S rRNA gene sequencing, a Softcup^®^ menstrual cup for cervicovaginal cytokine measurement (inserted for 30 min), and an endocercvical cytobrush sample for RNA-Seq analysis. Upon arrival at the laboratory, vaginal swabs and menstrual cup secretions were stored at −80°C until testing and the cytobrush sample was processed for RNA-Seq as described below. Blood was obtained for HIV rapid test (Determine™ HIV-1/2, Abbott) and herpes simplex virus 2 (HSV-2) serology (KALON). For all the participants, the sample collection was performed at the end of the contraceptive cycle just prior to starting a new contraceptive method, hence during the placebo phase/NET nadir. No samples were collected during menstruation, instead the visit was rescheduled.

### STI and BV Testing

Molecular testing for the STIs *Chlamydia trachomatis, Neisseria gonorrhoeae, Trichomonas vaginalis*, and *Mycoplasma genitalium* by multiplex PCR was performed on vulvo-vaginal swabs at each visit as described (42). If any of these laboratory-based tests were positive, appropriate targeted therapy was prescribed and recorded. Blood was obtained for HIV rapid test (Determine™ HIV-1/2, Abbott) and herpes simplex virus 2 (HSV-2) serology (KALON). A vulvo-vaginal swab was collected for BV testing [Gram staining and Nugent scoring; BV negative (Nugent 0–3), intermediate (Nugent 4–6), or positive (Nugent 7–10)] and microscopy for *Candida* hyphae and spores. Treatment for BV was only offered to participants presenting with clinical symptoms according to South African syndromic management guidelines. Vaginal pH was measured using color-fixed indicator strips (Macherey-Nagel, Düren, Germany).

### RNA-Seq Library Preparation

Endocervical cytobrush samples collected at crossover visit (*n* = 107) were processed for RNA-Seq (Supplementary Figure 1A). Cells were collected using a Digene cervical sampler (Digene Corporation, Gaithersburg, MD, USA) inserted into the endocervical os, rotated through 360°, placed in a tube containing 3 ml R10 media, and transported to the laboratory within 4 h. The cytobrush was rotated against the side of the tube to dislodge the cells and flushed with the media. The tubes were centrifuged at 1,200 rpm for 10 mins to pellet the cells. The supernatant was removed and the pellet lysed in 350 μl RLT buffer (QIAGEN) with 1% beta-mercaptoethanol and stored at −80°C. RNA was extracted from the endocervical cytobrush samples using RNeasy Micro kits and QIAcube automation. RNA quantity and quality were determined by Qubit and Bioanalyzer analysis, respectively. Due to the clinical nature of the samples, many exhibited a moderate level of degradation, and the average RNA integrity number score was 5. To prepare libraries, we used the Illumina TruSeq RNA Exome kit (Illumina Inc. San Diego, CA, USA), which employs random hexamer-based cDNA generation and enriches exonic sequences to mitigate the impact of RNA degradation. Briefly, libraries were prepared as per manufacturer's instructions, with 20 ng of total RNA as the input. The amplified libraries were validated by capillary electrophoresis on the Agilent 4200 TapeStation. Libraries were normalized, pooled, and sequenced on the Illumina HiSeq3000 system employing a single-end 101 cycles run at average read depths of 21 million reads/sample.

### RNA-Seq Mapping and Quality Control

RNA-Seq data was demultiplexed using the Illumina bcl2fastq version 2.20.0.422 BCL to FastQ file converter. The quality of raw reads was assessed with FastQC version 0.11.8 (43) (http://www.bioinformatics.babraham.ac.uk/projects/fastqc). Reads were mapped to the GRCh38 human genome assembly (https://www.ncbi.nlm.nih.gov/assembly/GCF_000001405.26/) using STAR version 2.5.2b with default alignment parameters (44). Abundance estimation of raw read counts per transcript was done internally with STAR using the htseq-count algorithm (45). A series of quality control metrics was evaluated to filter out low-quality samples that could falsely bias the gene expression data. Of the 107 libraries prepared, 96 samples were retained (Supplementary Figure 1B). Samples were retained for analysis based on the following criteria: (1) >5 M unique reads mapping to the host (Supplementary Figures 1C,D), (2) a median coefficient of variation (CV) coverage <1.3 (Supplementary Figure 1E), (3) a variance in relative log expression between −2 and 2 (Supplementary Figure 1F). For downstream analysis, only protein-coding probe-target genes were considered, and they were obtained as follows: Probe coordinates from the Illumina TruSeq RNA Exome kit were available for hg19 and were thus cross-mapped to hg38 genome assembly using cross-map, an assembly converter tool. Genes which are classified as “protein-coding” by the gene biotype field in the hg38 GTF file and for which the exon coordinates showed an overlap of at least one base with any of the probe coordinates, formed the set of protein-coding probe-target genes (19,543 genes). The overlap across the exon coordinates and probe coordinates was computed using the “closest” tool from bedtools. Additionally, lowly expressed genes were filtered out, i.e., genes with a mean raw count ≤5 across all samples, to yield a final set of 14,226 genes.

### Analysis of RNA-Seq Data

DESeq2 version 1.22.1 in Bioconductor/R platform was used to perform the differential expression analysis across the study arms (46). DESeq2 was run with a single-factor experimental design formula; design = ~assigned_contraceptive. The assigned contraceptive factor included three levels: Net-En, COC, and CCVR. Differential expression analysis results were extracted for cross-arm comparisons, i.e., CCVR vs. Net-En, CCVR vs. COC, and Net-En vs. COC. For these crossarm comparisons, the analysis was conducted as an intention-to-treat (ITT), in which participants were maintained in their originally assigned contraceptive groups to maintain a balance of random variables. As a validation check, the cross-arm comparisons were alternatively analyzed in a per-protocol (PP) design, which included only the participants who remained on the contraceptive method allocated at randomization, which removed a total of nine subjects compared to ITT analysis (changes to each group were: NetEn = 0; COC = −3; CCVR = −6) and the results were found to be congruent (Supplementary Figures 2, 3, Supplementary Tables 1, 2).

Additionally, although the rates of *N. gonorrhoeae* infection were evenly distributed between the treatment groups at enrolment, we observed an increased prevalence after the treatment phase in the CCVR arm. While the increased observation of *N. gonorrhoeae* may be a mediator of the treatment, to test the impact of this variable the cross-arm comparisons were reanalyzed with the subjects testing positive removed (changes to each group were: Net-En *n* = −1, COC *n* = −2, CCVR, *n* = −7), and the results were found to be consistent with the primary analysis (Supplementary Figure 4, Supplementary Tables 3, 4). The selection thresholds to define differentially expressed genes (DEGs) in each comparison were as follows: false discovery rate (FDR) <0.05, absolute fold-change (FC) >1.5, i.e., absolute log_2_ FC >0.58, and a standard error estimate for the log_2_ fold-change estimate (lfcSE) <1. The DEGs were analyzed using the DAVID functional annotation tool (47) with an enrichment score cut-off of 1.3 (EASE = 0.05). GSEA was performed on the regularized log expression data using the Broad Institute GSEA tool from Molecular Signature Database (MSigDB) (48) (http://www.broadinstitute.org/gsea/msigdb/index.jsp). Heatmaps were generated using the ComplexHeatmap package in R. Violin plots were used to plot read counts using ggplot2. The mixOmics package (49) was used for DIABLO analysis (50) for the integration of log-transformed 16S rRNA sequencing data, cytokine, and RNA-Seq data to identify correlated variables that best discriminate between the contraceptive methods.

### Amplification and Sequencing of the V4 Region of the 16S rRNA Gene

Microbiome analysis was described in detail previously (9). In brief, vaginal lateral wall swabs were thawed and treated with an enzyme cocktail. Microbial DNA was extracted using the *Quick*-DNA^TM^ Fungal/Bacterial Miniprep kit (Zymo Research). Mechanical disruption was performed in a Qiagen TissueLyser LT. The V4 hypervariable region of the bacterial 16S rRNA gene was amplified by PCR using modified universal primers (51). Samples were purified using Agencourt AMPure XP beads (Beckman Coulter, Brea, CA, United States). Illumina sequencing adapters and dual-index barcodes were added to the purified amplicon products using limited cycle PCR and the Nextera XT Index Kit (Illumina). Amplicons were pooled, and the resultant libraries were purified by gel extraction and quantified using the Qubit dsDNA HS Assay Kit (Life Technologies). The libraries were sequenced on the Illumina MiSeq platform (300 bp paired-end) with v3 chemistry.

### Bioinformatics Analysis of the 16S rRNA Gene Sequencing Data

Demultiplexed raw reads were preprocessed using usearch 7 (52). Primer sequences were removed and reads truncated at 250 bp. Sequences were dereplicated, sorted by abundance, and clustered *de novo* into operational taxonomic units (OTUs) at 97% similarity. Chimeric sequences were detected using UCHIME (53) and removed. Taxonomic assignment was performed in QIIME 1.8.0 (54) using the RDP classifier against the GreenGenes 13.8 reference taxonomy. To increase species-level resolution, we used the usearch_global command implemented in VSEARCH (55) to search the *de novo* picked OTUs' representative sequences against our own Custom Vaginal 16S Reference Database, as previously described (56, 57). All hits with ≥ 97% identity were accepted. The remaining OTUs (<97% identity) were manually curated. OTUs that mapped to more than one species (with the same identity score) were annotated as follows: if an OTU mapped to two or three species, the OTU would be named Genus speciesA_speciesB or Genus speciesA_speciesB_speciesC, respectively; if an OTU mapped to more than three species but one species was clearly associated with vaginal microbiota, the OTU was named Genus species_cluster, where “species” was selected based on the majority of hits (57). Samples with ≥ 5,000 reads were selected for downstream analyses. The OTU table was normalized and filtered so that each OTU had at least 10 counts in at least 20% of samples or a relative abundance of at least 0.001%.

### Cytokine Measurements

The concentrations of IL-1β, IL-4, IL-6, IL-10, IL-17A, IL-17F, IL-21, IL-22, IL-23, IL-25, IL-31, IL-33, IFN-γ, sCD40L, and TNF-α in Softcup^®^ cervical secretions were measured by Luminex Milliplex assay using the Bio-Plex Pro Human Th17 Cytokine Panel, Bio-Plex Suspension Array Reader (Bio-Rad Laboratories Inc., USA) and Bio-plex manager software version 4 (Bio-Rad Laboratories, Inc.) as previously described (5). A 5-parameter logistic regression formula was used to calculate sample concentrations from the standard curve, and samples with cytokine concentrations below or above the limit of detection were assigned half the lowest detectable value or the highest detectable value, respectively, for the specific cytokine. Specimens from six participants were run across all plates (interplate controls), and six samples were duplicated on each set of plates (intraplate controls) for quality control measures. Spearman's rank test was used to measure intraassay and interassay correlation coefficients to determine assay reliability and reproducibility. For the individual cytokines, a cut-off of 55% detectable samples was set for inclusion in analyses.

### Power Calculations

The sample size of the substudy was limited to that of the parent study, where power calculations were based on the parent study's primary outcome, the relative acceptability of combined contraceptive vaginal ring vs. other modalities (Net-En injectable and oral contraceptives) based on the total score for the ORTHO BC SAT questionnaire at 4 months after randomization (58). We, however, performed a retrospective power analysis for the transcriptomics data to show that a sample size of 30 in each study arm was sufficient to identify at least 60% of DEGs with counts lower than 10 reads and at least 80% of DEGs with counts more than 10 reads with an absolute fold-change of 1.5 (Supplementary Figure 5). The sequencing depth is at 21 million reads/sample, and FDR is controlled at 0.05. To compute the power-sample size relationship, PROPER, a simulation-based R package (59), was used. The estimation parameters were computed based on the normalized gene counts for all samples in the data set. The power-sample size relationship was computed on the differential expression estimation results obtained from DESeq2.

### Statistics

All downstream statistical analyses were performed in RStudio. Differences in study population characteristics according to study arm were tested using Pearson's Chi-squared test or Fisher's exact test (when the expected value was <5) for count data, unpaired Student's *t*-test for differences in mean (parametric data), and unpaired Mann-Whitney U test for differences in medians (nonparametric data).

### Data Availability

Transcriptomic data is available in the Gene Expression Omnibus (GEO) repository under accession number GSE171825, and raw sequence data for 16S rRNA gene amplicon sequences are available at http://www.ebi.ac.uk/ under project number PRJEB30774. Custom R scripts and supporting documentation on the RNA-Seq analyses are available at https://github.com/BosingerLab/Balle_etal_UCHOOSE_Frontiers_RNASeq.

## Results

### Cohort Characteristics

**Figure 1:**
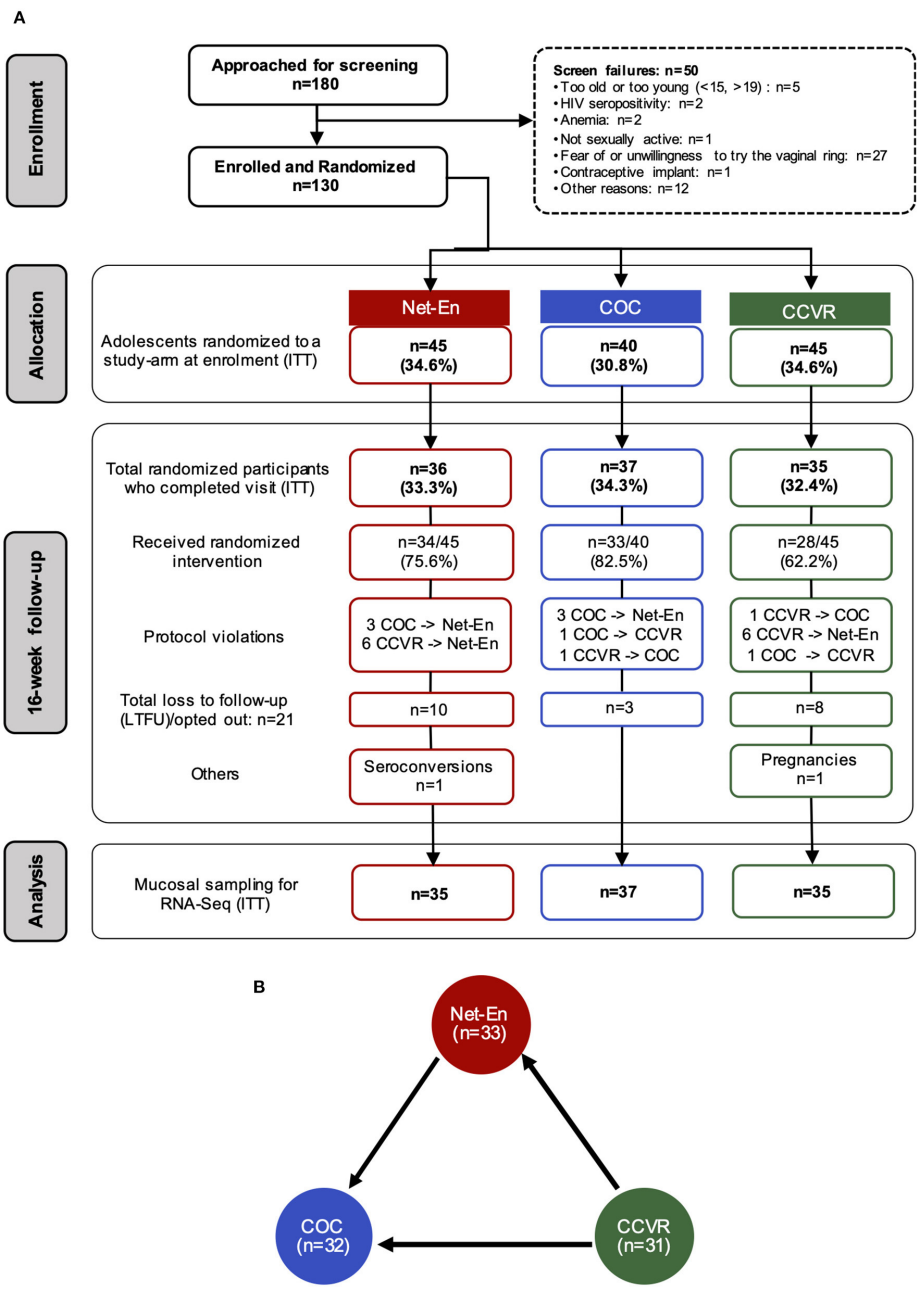
Study overview and randomization. **(A)** CONSORT diagram of the number of participants that completed each study visit and provided endocervical cytobrushes for RNA-Seq analyses, and the distribution of participants within each arm of study: Net-En injection, combined oral contraceptives (COC), and the vaginally inserted combined contraceptive ring (CCVR). ITT, intention-to-treat. **(B)** Number of samples that passed RNA-Seq quality filtering at the 16-week follow-up within each study arm. Differential expression analysis on the RNA-seq data was carried out for cross-arm comparisons, Net-En vs. COC, CCVR vs. Net-En, and CCVR vs. COC. Arrows indicate the RNA-Seq statistical comparisons used.

One hundred and eighty adolescent females were screened for the parent study (NCT02404038) (41), and 130 were enrolled (Supplementary Table 5) in the current substudy. Of these, 45 were randomized to CCVR, 45 to Net-En, and 40 to COC (Figure 1A) (9). At baseline, adolescents in each study arm were similar in demographics and reported sexual behavior, medical, and reproductive history [including age, body mass index [BMI], vaginal insertion practices, and antibiotic use, and also bacterial vaginosis (BV), *Candidiasis*, and STI prevalence] (41) (Supplementary Table 6). The majority of participants (99/130, 76%) were using a HC method before enrolment, and no washout period was introduced. However, the distribution of prior HC use was similar across study arms at baseline (Supplementary Table 6). A total of 107 adolescents reached the 16 week follow-up visit and provided an endocervical cytobrush sample (Net-En, *n* = 35; COC, *n* = 37; CCVR, *n* = 35) for RNA-Seq analysis (Figure 1A).

### CCVR Use Induces Greater Endocervical Transcriptional Perturbations Compared to COC and Net-En Injections

After quality filtering, RNA-Seq data from 96 participants (Net-En, *n* = 33; COC, *n* = 32; CCVR, *n* = 31) were considered suitable for differential gene expression analysis (Figure 1B, Supplementary Figure 1). To test if HC methods had distinct effects on the FRT transcriptome, we conducted a cross-sectional ITT analysis 16 weeks after contraceptive initiation. At this visit, the participants in the three study arms did not significantly differ in BV (by Nugent score), *Candida*, or overall STI prevalence, HSV-2 serology, antibiotic use, or sexual risk behavior (Supplementary Table 7). However, as described previously, the prevalence of *N. gonorrheae* was higher among CCVR users, and adolescents assigned to COC had more optimal bacterial communities and adolescents assigned to CCVR generally had higher levels of inflammatory cytokines (Supplementary Table 7) (9, 40).

**Figure 2:**
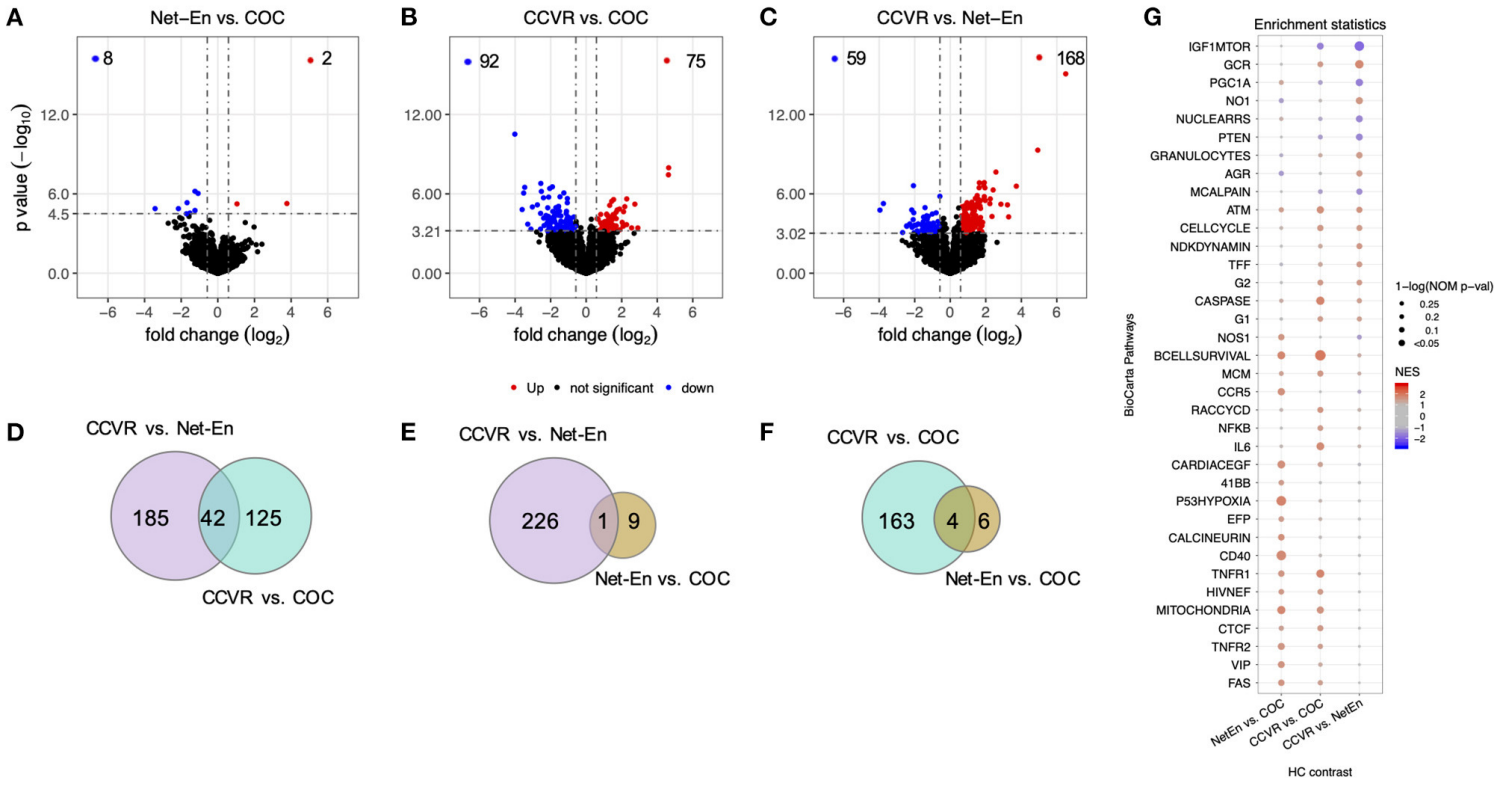
CCVR leads to greater alterations of the endocervical transcriptome compared to COC and Net-En injectable. The results of the statistical analyses on differential expression of cross-arm comparisons, namely Net-En vs. COC, CCVR vs. COC, and CCVR vs. Net-En, have been shown by the different panels (Net-En: *n* = 33, COC: *n* = 32, CCVR: *n* = 31). The significantly differentially expressed genes (DEGs) were identified by the following criteria: false discovery rate (FDR) <0.05, absolute fold-change >1.5, and standard-error in fold-change (lfcSE) <1. **(A–C)** Volcano plots showing statistical significance against fold-change for each comparison, with the DEGs highlighted in red (upregulated) and blue (downregulated) colors indicated in the upper quadrants. **(D–F)** Venn diagrams showing the numbers of overlaps of DEGs across the comparisons. **(G)** Dot plot showing BioCarta pathways enriched in at least one of the three comparisons (nominal *p* < 0.1). The pathways are ordered by the nominal *p*-value of CCVR vs. Net-En contrast, with the most-significant pathway shown at the top. The normalized enrichment scores are represented by a blue to red color gradient, blue for negative scores and red for positive scores. The significance of the enrichment of that pathway is represented by the size of the dot [1-ln (nominal *p*-value)].

We assessed DEGs between each pair of study arms in a primary analysis of randomized treatment groups (i.e., Net-En vs. COC, CCVR vs. Net-En, and CCVR vs. COC) using DESeq2 (Figure 2, Supplementary Table 8). After 16 weeks of assigned contraceptive use, women in the Net-En and COC arms showed very limited differences in gene expression with only 10 DEGs identified (Figure 2A). In contrast, 167 DEGs were identified between the CCVR and COC arms (Figure 2B) and 227 between CCVR and the Net-En arms (Figure 2C), indicating a stronger impact of CCVR relative to the systemic HCs. No DEGs were shared between all three comparisons (Figures 2D–F).

### Immune Pathways Were More Highly Expressed in the CCVR Compared to Net-En and COC Arms

To identify enriched pathways in a large number of DEGs identified between CCVR and the Net-En and COC arms (Figures 2B,C), we performed two complementary analyses. First, we identified functional annotation clusters using the Database for Annotation, Visualization, and Integrated Discovery (DAVID) (47) (Table 1, Supplementary Table 9). Comparison of the 168 DEGs upregulated in CCVR relative to Net-En arm revealed 50 significantly enriched functional clusters. The majority of these clusters were associated with immune signaling (Table 1, Supplementary Table 9). Analysis of DEGs upregulated in the CCVR arm compared to COC yielded 9 clusters with functions related to cell cycle control, inflammation, and negative regulars of metabolism (Table 1).

**Table 1:** Annotation clusters enriched in the CCVR arm relative to the Net-En and COC arm.

**Annotation cluster**	**Representative annotation terms**	**Enrichment score**
**CCVR vs. Net-En**		
1	Regulation of immune response	9.21
2	Innate immune response	8.37
3	Response to stimulus	5.21
4	Regulation of inflammatory response	4.68
5	Negative regulation of immune response	4.41
6	Cytokine production	4.40
7	Regulation of signal transduction	4.37
8	Adaptive immune response	4.28
9	Apoptotic process	4.10
10	Response to biotic stimulus	4.09
11	Response to mechanical stimulus	3.69
12	Cellular process	3.38
13	Pattern recognition receptor signaling pathway	3.29
14	Interleukin-2 production	3.17
15	Immune cell migration	3.12
16	Type I interferon production	3.08
17	Programmed cell death	3.03
18	Response to interferon-gamma	2.93
19	Interleukin-1 production	2.91
20	Cell motility	2.88
**CCVR vs. COC**		
1	Cell cycle process	4.72
2	Chromosome organization	3.93
3	Meiotic cell cycle process	2.38
4	Negative regulation of cellular process	2.03
5	Interleukin-1 production	1.99
6	Regulation of cell cycle	1.97
7	Lymphocyte activation	1.93
8	Cytokine/chemokine production	1.59
9	Interspecies interaction	1.36

*The differentially expressed genes (DEGs) for each pair of study armscomparisons were analyzed using the DAVID Functional Annotation Clustering Tool. Top annotation clusters with enrichment scores less than or equal to 0.05 (equivalent to 1.3 in minus log scale) have been included (max. 20) and ordered by group enrichment score. The representative biology terms associated with the annotation clusters were manually selected. CCVR, combined contraceptive vaginal ring; COC, combined oral contraceptives*.

**Figure 3:**
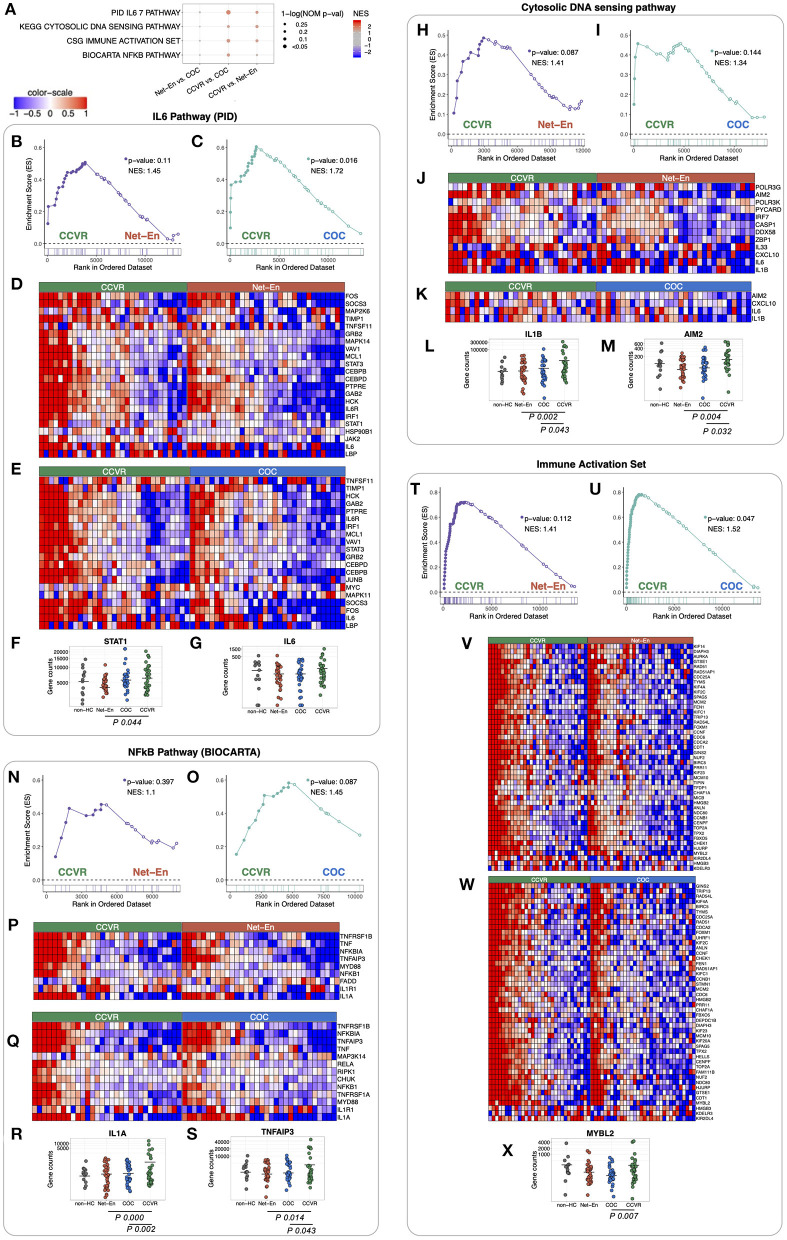
Pathways associated with immune responses and DNA repair enriched in CCVR arm compared to Net-En and COC (nominal *p* < 0.1). Enrichment line plots, leading-edge gene heatmaps, and plots of gene counts have been shown for both CCVR vs. Net-En as well as CCVR vs. COC comparisons for the enriched pathways. **(A)** A dot plot to represent the gene set enrichment analysis (GSEA) statistics for the enriched pathways. The statistical significance of the enriched pathways is shown by the size of the dots [1-ln (nominal *p*-value)], and the normalized enrichment score (NES) is represented by a blue-to-red color-gradient, blue for negative scores and red for positive scores. **(B,C,H,I,N,O,T,U)** In the enrichment line plots, the running enrichment score (y-axis) is indicated for each gene ordered by their rank in the whole data set for that specific comparison, shown by the vertical bars shown below the x-axis. **(D,E,J,K,P,Q,V,W)** Heatmaps for the leading-edge genes of the indicated pathways. The gene expressions are log-transformed, further normalized by mean of all samples in the two study arms, in each comparison. The color gradient goes from blue to red colors in representing the lowest to the highest gene expression across all samples in the comparison. **(F,G,L,M,R,S,X)** For a couple of leading-edge genes, the distribution of the normalized counts in each study arm has been shown. The mean counts within each study arm are represented by a horizontal bar. If the gene is significantly differentially expressed (p-adj < 0.05) then it's p-adj has been shown below the counts' plot.

We next performed gene set enrichment analysis (GSEA) using various well-defined gene-set/pathway collection databases from the Molecular Signatures Database (https://www.gsea-msigdb.org/gsea/msigdb) (48, 60–64) and for custom in-house gene-sets that include Serpins, Integrins, and immune response genes (30, 65–69) (Figure 2G, Supplementary Tables 10, 11). In concordance with the cluster analysis, pathways associated with response to cellular stress and external stimuli, including inflammatory, apoptotic, and DNA repair pathways, were enriched in the CCVR arm compared to both the Net-En and COC arms (Figure 2G, Supplementary Figure 6). These included the IL-6 pathway (Figures 3A–E) with genes such as STAT1 (Net-En vs. CCVR: P adj. = 0.044) being significantly upregulated (Figure 3F) and the IL-6 gene itself albeit not significantly so (Figure 3G). The DNA sensing pathway was also enriched in the CCVR arm (Figures 3H–K) with genes including IL1B (Net-En vs. CCVR: P adj. = 0.002, COC vs. CCVR: P adj. = 0.043) and AIM2 (Net-En vs. CCVR: P adj. = 0.004, COC vs. CCVR: P adj. = 0.032) being significantly upregulated (Figures 3L,M). So was the NFkB pathway (Figures 3N–Q) with significantly upregulated genes such as IL1A (Net-En vs. CCVR: P adj. <0.0001, COC vs. CCVR: P adj. = 0.002) and TNFAIP3 (Net-En vs. CCVR: P adj. = 0.014, COC vs. CCVR: P adj. = 0.043) (Figures 3R,S). Accordingly, custom in-house gene-sets associated with inflammation and immune activation were more highly expressed in the CCVR arm than both the Net-En and COC arms (Figures 3T–W, Supplementary Figure 7) which included significantly DEGs such as IL1B, AIM2, IL1A, TNFAIP3, MYBL2 (COC vs. CCVR: P adj. = 0.007) (Figure 3X).

### Net-En Use Associated With Increased Keratinization and Cell Migration

**Figure 4:**
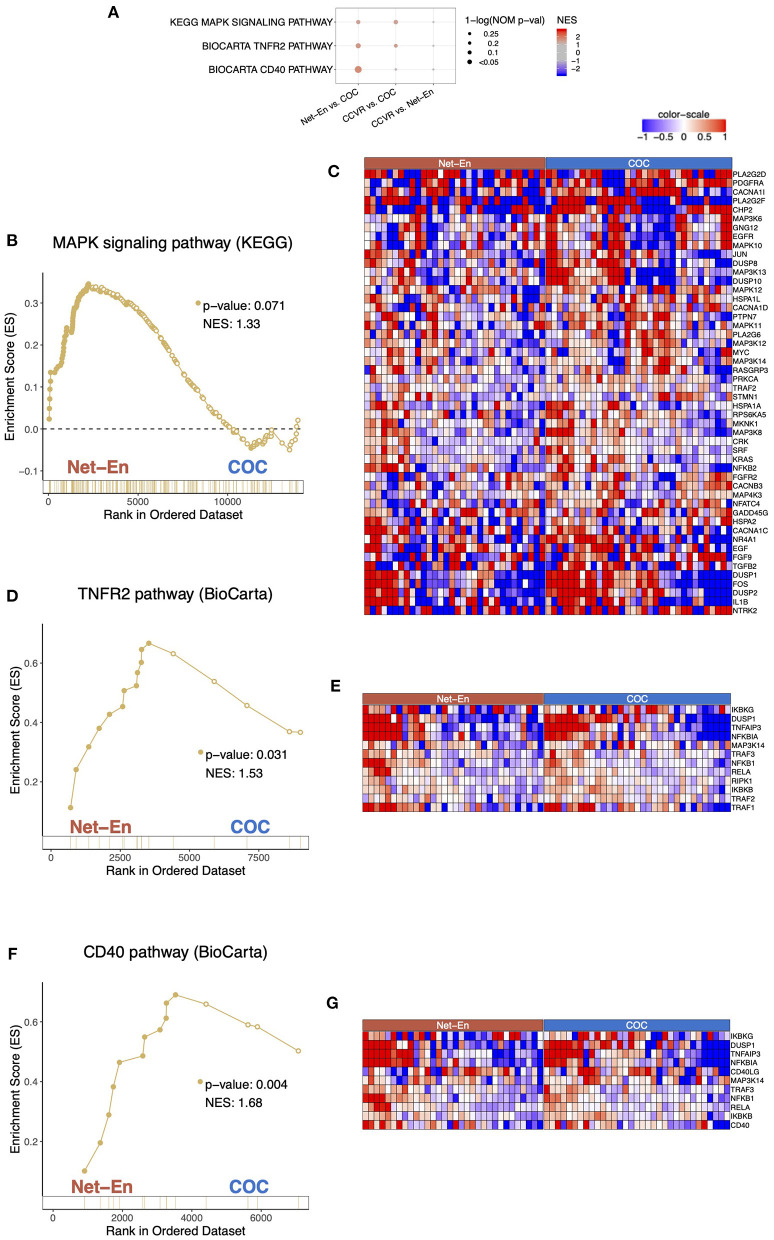
Pathways associated with cell migration were enriched in the Net-En arm (nominal *p* < 0.1). Enrichment line plots, leading-edge gene heatmaps, and plots of gene counts have been shown for Net-En vs. COC comparison for the enriched pathways. **(A)** A dot plot to represent the gene set enrichment analysis (GSEA) statistics for the enriched pathways. The statistical significance of the enriched pathways is shown by the size of the dots [1-ln (nominal p-value)], and the normalized enrichment score (NES) is represented by a blue-to-red color-gradient, blue for negative scores and red for positive scores. **(B,D,F)** In the enrichment line plots, the running enrichment score (y-axis) is indicated for each gene ordered by their rank in the whole data set for that specific comparison, shown by the vertical bars shown below the x-axis. **(C,E,G)** Heatmaps for the leading-edge genes of the indicated pathways and comparisons. The gene expressions are log-transformed, further normalized by mean of all samples in the two study arms. The color gradient goes from blue to red colors in representing the lowest to the highest gene expression across all samples in the comparison.

Cluster analysis of the 59 DEGs significantly upregulated in participants in the Net-En arm compared to the CCVR arm revealed 6 clusters (ES 2.83 to 1.59) that were mainly associated with keratinization, epithelial differentiation, and cell motility (Table 2, Supplementary Table 9). Furthermore, GSEA pathways associated with cell–cell adhesion and migration were higher in the Net-En arm compared to the CCVR arm (Figure 2G, Supplementary Table 11, Supplementary Figure 6) and pathways associated with signal transduction (e.g., MAPK signaling) were also enriched in the Net-En arm (Figures 4A–C). TNF signaling pathways were enriched in the Net-En arm compared to both the CCVR and COC arms (Figures 4D,E). Of the 10 DEGs observed between the Net-En and COC arm, 8 genes were downregulated in the Net-En arm and of these, three were negative regulators of type I interferon (IFN) antiviral immune responses [IFI44L (P adj. = 0.045), SIGLEC1 (P adj. = 0.016), and USP18 (P adj. = 0.032)] (Supplementary Table 8). Consistent with this finding, a pathway comprised of interferon stimulating genes (ISGs) trended toward downregulation in the Net-En arm was compared to the CCVR arm and the COC arm, although no comparison was significant (Supplementary Figure 8). Pathways upregulated in both CCVR and Net-En vs. COC or in CCVR and COC vs. Net-En were uncommon (Supplementary Figure 6). Yet, we noted that certain pathways associated with B cell signaling were enriched in the Net-En and CCVR arms vs. the COC arm and compared to controls (Figures 4F,G).

**Table 2:** Annotation clusters enriched in the Net-En or COC arms relative to the CCVR arm.

**Annotation cluster**	**Representative annotation terms**	**Enrichment score**
**Net-En vs. CCVR**		
1	Keratinization	2.83
2	Epithelial cell differentiation	2.18
3	Drug metabolism	2.10
4	Cell motility/angiogenesis	2.06
5	Signal peptide	1.92
6	Cell death/terminal differentiation	1.59
**COC vs. CCVR**		
1	Keratinization	3.76
2	Cellular response to inorganic substance	2.47
3	Establishment of skin barrier/water homeostasis	2.37
4	Lectin	1.95
5	Carbohydrate biosynthesis	1.62

*The differentially expressed genes (DEGs) for each pairwise comparison were analyzed using the DAVID Functional Annotation Clustering Tool. Annotation clusters with enrichment scores less than or equal to 0.05 (equivalent to 1.3 in minus log scale) have been included and ordered by group enrichment score. The representative biology terms associated with the annotation clusters were manually selected. CCVR, combined contraceptive vaginal ring; COC, combined oral contraceptives*.

### COC Initiation Associated With Increased Keratinization Relative to CCVR and Enrichment of Metabolic Pathways

**Figure 5:**
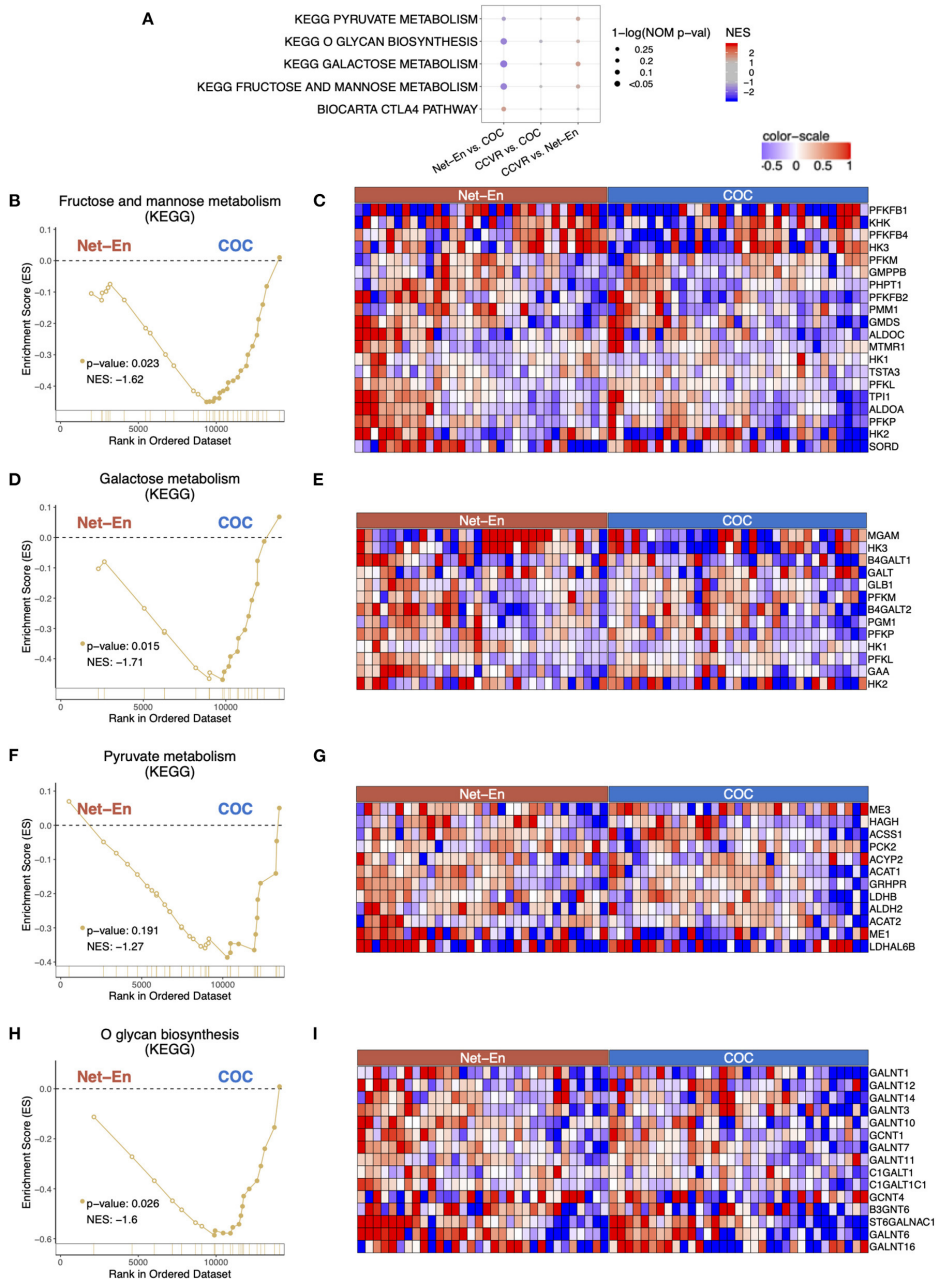
Pathways associated with metabolism were enriched in the COC arm. Enrichment line plots, leading-edge gene heatmaps, and plots of gene counts have been shown for Net-En vs. COC comparison for the enriched pathways. **(A)** A dot plot to represent the gene set enrichment analysis (GSEA) statistics for the enriched pathways, The statistical significance of the enriched pathways is shown by the size of the dots [1-ln (nominal *p*-value)] and the normalized enrichment score (NES) is represented by a blue-to-red color-gradient, blue for negative scores and red for positive scores. In keeping the order of the comparison consistent throughout the manuscript, the results are reported in the order Net-En vs. COC. **(B,D,F,H)** In the enrichment line plots, the running enrichment score (y-axis) is indicated for each gene ordered by their rank in the whole data set for that specific comparison, shown by the vertical bars shown below the x-axis. **(C,E,G,I)** Heatmaps for the leading-edge genes of the indicated pathways and comparisons. The gene expressions are log-transformed, further normalized by mean of all samples in the two study arms. The color gradient goes from blue to red colors in representing the lowest to the highest gene expression across all samples in the comparison.

Cluster analysis of the 92 genes with higher expression levels in the COC arm compared to the CCVR arm revealed 5 clusters associated with keratinization, epithelial barrier integrity, and carbohydrate metabolism (Table 2, Supplementary Table 9). Furthermore, GSEA pathways associated with cell–cell adhesion and migration were higher in the COC arm compared to the CCVR arm (Supplementary Table 11, Supplementary Figure 6) and pathways related to hexose (Figures 5A–E), pyruvate metabolism (Figures 5F,G), and glycosylation (Figures 5H,I) were enriched in COC vs. Net-En.

### Integration of Cervicovaginal Transcriptomics, Cytokine, and Microbiome Data

**Figure 6:**
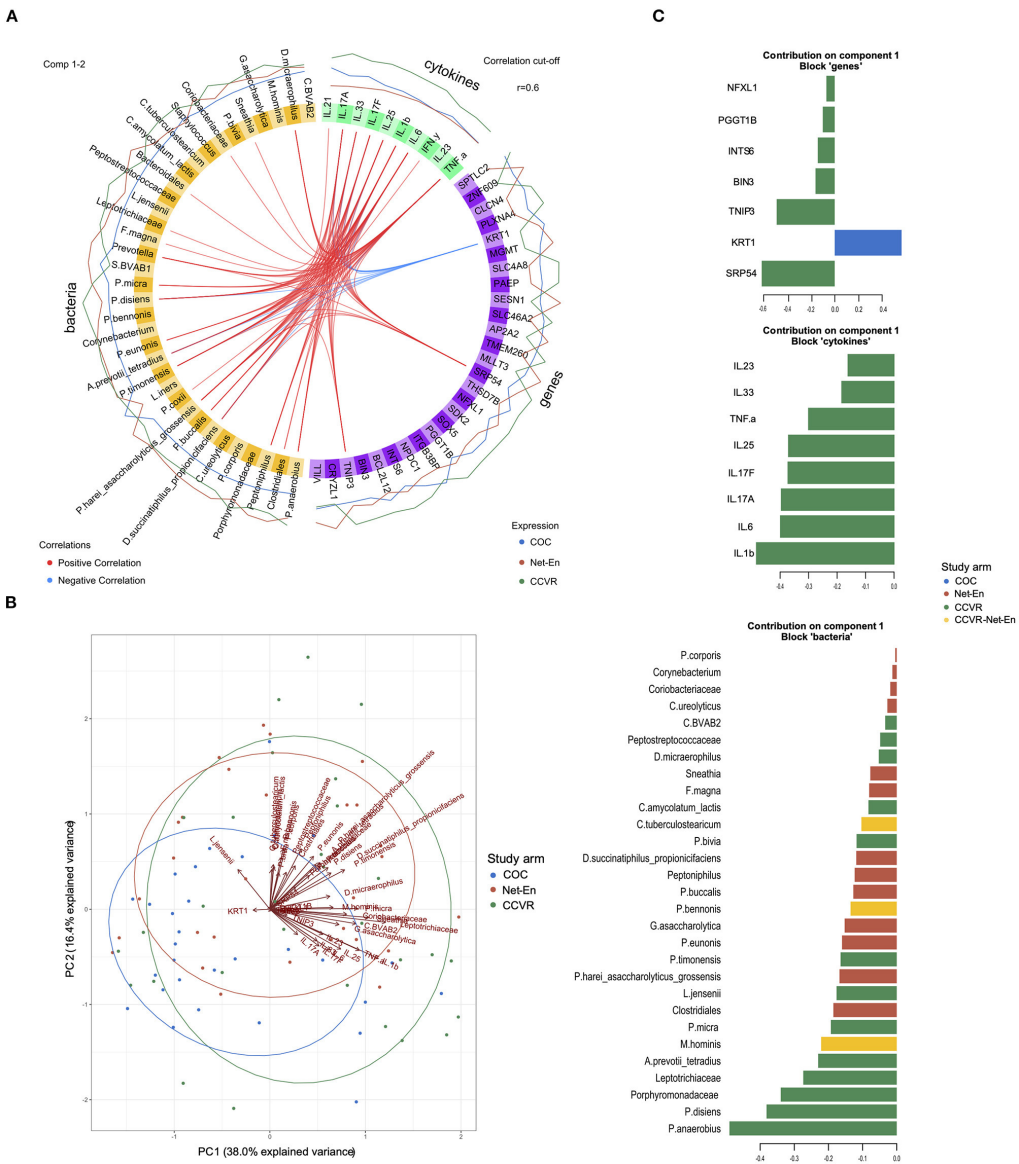
Integration of gene expression, microbiome, and cytokine data. **(A)** Circos plot depicting correlated bacteria (orange), cytokines (green), and genes (purple) identified using DIABLO analysis as most discriminatory between the study arms. The e xpression level of each variable is indicated by the lines outside the circle and colored according to each study arm (Net-En: red, combined oral contraceptives (COC): blue; combined contraceptive vaginal ring (CCVR): green). Positive (red) or negative (blue) correlations between individual variables are indicated with lines connecting these within the circle. Only *R*^2^ values > 0.6 are displayed. **(B)** Biplot for principal components measured for the variables (genes, cytokines, and bacteria) identified in the DIABLO analysis (component 1) as discriminatory between the study arms. **(C)** Barplot showing the loadings of component 1 for each group of variables (genes, cytokines, and bacteria) from the DIABLO analysis. Each bar length corresponds to the loading weight (importance) of the individual gene, cytokine, or bacteria. The colors corresponds to the study arm in which the feature contributes the most (red: Net-En; blue: COC; green: CCVR; yellow: contributing equally to CCVR and Net-En arms).

We have previously reported that adolescents randomized to COCs in this cohort had significantly lower vaginal microbiota diversity and lower relative abundance of taxa associated with HIV risk compared to those who initiated Net-En or CCVR, and that participants randomized to CCVR showed increased levels of proinflammatory and Th17-related cytokines (9). To identify correlated variables (genes, cytokines, and bacteria) best discriminating between the study arms, we used data integration analysis for biomarker discovery using latent variable approaches for “Omics” studies (DIABLO) multivariate analysis (50) (Figure 6). We confirmed that all 10 cytokines included in the DIABLO model (i.e., IL-1β, IL-17A, IL-17F, IL-6, IL-25, TNF-α, IL-33, IL-23, IFN-γ, and IL-21) were present at higher levels in the CCVR arm (Figure 6). These cytokines were all positively correlated with several BV-associated bacteria (which were generally more abundant in the CCVR and Net-En arms compared to the COC arm) (Figure 6A). These cytokines and bacteria were all positively correlated with genes associated with cell proliferation and inflammation (TNIP3, PGGT1B, BIN3, NFXL1, INTS6, and SRP54) (Supplementary Figure 9), all of which were expressed at a higher level in the CCVR arm (Figure 6A). In contrast, these cytokines and bacteria showed a strong negative correlation with KRT1, a gene from the keratin gene family associated with differentiation of epithelial tissues, which was expressed at the highest level in the COC arm (Figure 6). Additionally, the proinflammatory cytokines IL-6, IL-1β, IFN-γ, and TNF-α also showed negative correlation with SESN1 and ITGB3 (Supplementary Figure 9). SESN1 was more highly expressed in the Net-En arm, and ITGB3, an integrin involved in cell adhesion, was more highly expressed in the COC arm.

Th17-related cytokines were positively associated with genes functioning in regulating immune cell apoptosis and proliferation (BCL2L12, SOX5), supporting antiviral CD8+ T cell metabolic fitness (SPTLC2) (70), angiogenesis/metastasis of tumors (PLXNA4, CLCN4) and with genes with limited functional characterization (SDK2, SLC46A2/8, THSD7B, TMEM260) (all with highest expression levels in the CCVR arm) and negatively associated with SESN1, AP2A2, MLLT3, MGMT, and ZNF609 (all with highest expression in Net-En arm) (Figure 6). AP2A2, MGMT, and ZNF609 were negatively associated with taxa such as *Staphylococcus, C tuberculostearicum, P. anaerobius, P. bivia, P. disiens, L. jensenii*, and *L. iners*, which were less abundant in the Net-En arm and positively associated with *Prevotella*, BVAB-1, and *Bacteriodales*, which were more abundant in the Net-En arm. The inverse relationship between genes and bacteria was found for genes more highly expressed in the CCVR arm, including THSD7B, PLXNA4, CLCN4, SLC46A2/8, NPDC1, SOX5, and SDK2 (Figure 6). The loadings that contributed most to PC1 included *Prevotella disiens, Peptostreptococcus anaerobius*, IL-1β, IL-6, and SRP54, all higher in CCVR and KRT1, which was more highly expressed in the COC users (Figure 6C). Collectively, these integrated analyses allowed us to assess the impact of differing HCs on the FRT at a systems level and compare the quantitative influence within the microbiome, cytokine, and gene expression measurements taken together. This demonstrates that the most differentiating signal for CCVR use is consistent with elevated cervicovaginal inflammation, and that the strongest signal in COC is consistent with increased keratinization.

## Discussion

Young African women are at a high risk for both STIs and unintended pregnancies and are in need of safe and effective HCs. The impact of varying HCs on the mucosal immune environment has not been studied in detail using randomized designs, which overcomes biases introduced by observational data. In this study, we examined the effects of three HCs with different hormones and delivery strategies, including both combined and progestin-only methods, on endocervical gene expression of South African adolescents within a prospective randomized trial using high throughput transcriptomics. We found that adolescents randomized using the CCVR had significantly elevated expression of genes and pathways associated with immune responses compared to adolescents assigned to the COC and Net-En arms. These results affirm data from the same cohort in which we observed that CCVR users had significantly elevated inflammatory- and Th17-related cytokine concentrations in cervicovaginal fluid (CVF) compared to COC and Net-En users and relative to baseline levels (5, 9). Similarly, in a small prospective study of US women initiating CCVR (NuvaRing^®^, Merck), the endocervical expression of immune-related genes increased after 1 month of CCVR use compared to both the follicular and luteal phase of the menstrual cycle prior to CCVR initiation (71). We further observed that CCVR users had lower levels of transcriptional responses indicative of epithelial differentiation and keratinization relative to those assigned to Net-En or COC. While there is limited data on the effects of CCVRs on cervical gene expression, some studies have identified increased levels of genital matrix metalloproteinase (MMPs) and reduced levels of MMP tissue inhibitors (TIMPs) with CCVR initiation (71), suggestive of mucosal barrier repair or remodeling (18). Whether CCVR use mediates the disruption of epithelial barrier or whether this occurs up- or downstream of the CCVR-mediated increase in inflammation and dysbiosis observed in this cohort is yet to be determined. Regardless of the underlying mechanism, these findings demonstrate broad immune shifts following CCVR usage.

Several vaginal rings containing microbicides against HIV are currently in clinical trials and show promising results. In a recent exploratory analysis of MTN-023, a Phase IIa study of the dapivirine (DPV)-containing vaginal ring in American adolescents, and aproteomics analysis of the influence of the DPV ring on the mucosal proteome and microbiome showed only limited changes to host inflammatory pathways and the vaginal microbiome (72). Furthermore, in a small, placebo-controlled randomized trial, in which women were randomized to an intravaginal ring delivering either the antiretroviral drug tenofovir (TFV) alone or together with levonorgestrel (LNG) or a placebo, no change in epithelial thickness and microbiota was observed, but only minor cytokine changes were noted after approximately 15 days of use (73, 74). On the other hand, intravaginal rings made of hydrophilic elastomer HydroThane AL 25–93A tubing delivering 360 mg TFV disoproxil fumarate were previously shown to cause mucosal ulceration, not present in the placebo ring group, although the control group was small (*n* = 5) (75). Likewise, in the 1990s, the clinical trial of a LNG-releasing contraceptive vaginal ring trial was halted due to local lesions that developed after a median of 4 months of use (76).The differences between all these results may be attributable to differences in ring formulation and the size of the ring, the active compounds they deliver, dosing, time since ring initiation, sample collection, analysis approach, and study population. The latter may include the familiarity and comfort of participants with intravaginal products, which was low in this adolescent cohort (41) and could have contributed to the immune response observed with ring use.

Adolescents assigned to Net-En use had increased expression of genes associated with epithelial barrier integrity and reduced expression of genes associated with inflammatory responses relative to CCVR. Furthermore, there was a trend toward suppression of type I IFN stimulated genes in the Net-En arm compared to the other study arms. These results are somewhat consistent with our prior findings in which DMPA administration to pig-tailed macaques elicited a significant reduction in ISGs in cervical biopsies (30); however, the impact of Net-En on the ISG response observed in this study was milder. This may be partially due to DMPA being at an effectively elevated dose in our NHP studies due to more frequent injection (29, 30). Yet, differences in the metabolism and pharmacokinetics of Net-En vs. DMPA may also contribute to these differences in the ISG response.

In recent years, significant attention has been paid to the impact of progestin-only injectable contraceptives on the FRT mucosal environment, primarily DMPA-IM, but also Net-En, summarized in a recent extensive review by Ayele et al. (77). Our observation of enrichment of genes associated with keratinization and epithelial repair in subjects is consistent with a genital injury response, as reported by Birse et al. (22). Many of the more recent studies of longitudinal DMPA-IM use have reported, in general, a decrease in inflammatory cytokines (21, 78, 79); however cross-sectional studies have reported increased levels of macrophage inflammatory protein-3 alpha (MIP-3α)/CCL20 (80). In mechanistic studies in which cervical immune cell populations were prepared for transcriptomics, Byrne et al. observed that within the antigen-presenting cell (APC) population, pathways associated with inflammation were increased from DMPA-IM usage (81). While we did not observe widespread upregulation of pathways associated with inflammation, we did observe upregulation of the TNF-receptor signaling in subjects randomized to Net-En, suggesting that Net-En, similar to DMPA-IM, may result in increased inflammation within cervical APCs.

In recent mouse studies, treatment with different progestins including DMPA, LNG, and Net-En led to reduced genital levels of the cell–cell adhesion molecules, increased genital mucosal permeability, and increased susceptibility to genital pathogens (82, 83). These effects were, however, more pronounced in DMPA-vs. Net-En-treated mice. As such, different progestins may have differential effects on the FRT ecosystem, which may explain the differences observed between the studies (84–86). Of note, as progestin-mediated loss of genital epithelial integrity and barrier function may be dose-dependent (21, 35), the timing of mucosal sampling may influence the results. In this study, samples were collected after 2 months of the latest Net-En injection at a time of NET concentration nadir. Analysis of the impact of Net-En at multiple time points after injection as well as a head-to-head comparison of DMPA-IM and Net-En should be conducted in the future. Nevertheless, the results from this study suggest that Net-En is safe to use for adolescents at the risk of vaginal infections.

In this cohort, the use of COC was associated with increased epithelial barrier integrity, carbohydrate metabolism and glycosylation, and decreased inflammation relative to CCVR use. In a recent observational study by Zalenskaya et al., gene expression of the ectocervical mucosa of women initiating COC showed limited changes in gene expression, and, in contrast to DMPA-IM, did not cause significant alterations in the expression of genes responsible for mucosal barrier function (23). Similarly, in a recent cross-sectional study conducting transcriptional profiling of endometrial and cervical biopsies from women using hormonal or nonhormonal intrauterine devices, COCs or no-HC method, COC use showed minor effects on the endocervical and cervical transcriptome compared to controls (87). In a crosssectional study comparing DMPA-IM, LNG-implant, and COC, women using COC showed some degree of glycomic change in comparison to non-HC users and significantly higher levels of glycosylation of CVF proteins than DMPA-IM users (88). Glycosylation of CVF proteins, such as mucins, plays a critical role in their immunological functions, and the CVF coating of the cervical epithelium is an important immunological mediator, providing a barrier to infection (89, 90). Together with the results of this study, these studies suggest that the use of COC may be associated with protection against HIV infection through increased epithelial barrier stability and lack of inflammation.

Due to the randomized design, we were poised to overcome many of the challenges that have plagued prior observational studies that were confounding due to reductions in condom use by women using more effective contraceptives. For this reason, we performed an ITT analysis as our primary analysis to take advantage of the power of randomization. However, ~10% of adolescents did switch methods before the 16-week follow-up visit, and since the study was not blinded, the adolescents knew which HC method they were using, and therefore may have changed their behavior due to perceived risk for pregnancy with use of different and potentially unfamiliar HC methods. Yet, reported sexual risk behavior did not differ between the study arms at the 16-week follow-up visit. Furthermore, a PP analysis of the data showed similar results to the ITT analysis. In our study, adherence to the randomization arm tended to be poorest for those using COC, followed by the vaginal ring based on self-report (41); however, the adolescents in this study were actively seeking effective contraception and knew they had the risk of pregnancy with nonadherence. Some participants left the CCVR in for the fourth week (continuous use regimen) for the nurse to assist with ring removal, resulting in lack of withdrawal bleed. There was no significant difference in the time since the last menstrual period between the participants assigned to the CCVR and those to COC at the 16-week follow-up visit (9). Our study did not include a HC-unexposed group, and only a few participants were noncontracepting at baseline. Since these adolescents were sexually active and at risk of unintended pregnancies, inclusion of a washout period prior to assigned HC initiation was not possible. As such, previous contraceptive choice could have confounded results (with the majority of adolescents previously using Net-En). However, randomization ensured that there was equal distribution between arms of previously used methods of HC. Additionally, although the prevalence of *N. gonorrhoeae* were balanced between study arms at enrolment, we did observe an increase in subjects randomized to the CCVR treatment; however reanlaysis with these subjects removed did not impact the observation of increased pathways associated with inflammation in this group. Finally, our study is limited by the number of participants enrolled including the loss-to-follow-up rates (41) and lack of longitudinal samples for the participants. Future randomized trials with a larger number of contraceptive-naïve adolescents and a longitudinal comparison of the pre- and post-HC transcriptomic profile would be helpful to further study the biological impact of HC on FRT transcriptome.

In conclusion, this study encompasses one of the largest datasets to date describing the cervicovaginal immune system in response to HCs using systems-level approaches. These data allow a deeper understanding of the impact of commonly prescribed HCs on vaginal immunity and provide a basis for the development of improved administration of contraception to young women in HIV prevalent settings.

## Data Availability Statement

The datasets presented in this study can be found in online repositories. The names of the repository/repositories and accession number(s) can be found in the article.

## Ethics Statement

The parent study was approved by the Division of AIDS and the University of Cape Town Health Science Research Ethics Committee (HREC 801/2014) and was conducted in full compliance with South African Good Clinical Practice (SA-GCP), ICH76 GCP, and ICMJE guidelines. Approval for this substudy was obtained from the Human Research Ethics Committee at the University of Cape Town (HREC 801/2014). Written informed consent to participate in this study was provided by the participants' legal guardian/next of kin.

## Author Contributions

HJ, J-AP, CB, and SB conceived and designed the experiments. L-GB, KG, and SB designed and recruited the UChoose cohort. CB, SJ, IK, and A-UH processed samples and performed wet-lab experiments. HJ, J-AP, and L-GB acquired funding. CB, PG, GT, KL, and SB development of methodology. CB and PG formal analysis and visualization. CB, PG, HJ, and SB wrote the original draft of the manuscript. A-UH, KL, KG, SB, SJ, GT, IK, J-AP, and L-GB reviewed and edited the manuscript. All authors contributed to the article and approved the submitted version.

## Funding

This study was supported by the South African MRC and National Institutes of Health (NIH) grant R01 HD083040 (sub-study) and R01AI094586 (parent study). The Yerkes NHP Genomics Core was supported in part by NIH grant P51 OD011132 and S10 OD026799. CB was supported in part by the Poliomyelitis Research Foundation of South Africa grant 17/43.

## Conflict of Interest

The authors declare that the research was conducted in the absence of any commercial or financial relationships that could be construed as a potential conflict of interest.

## Publisher's Note

All claims expressed in this article are solely those of the authors and do not necessarily represent those of their affiliated organizations, or those of the publisher, the editors and the reviewers. Any product that may be evaluated in this article, or claim that may be made by its manufacturer, is not guaranteed or endorsed by the publisher.
