# Supplementary material for: Systems Analysis Reveals Contraceptive-Induced Alteration of Cervicovaginal Gene Expression in a Randomized Trial
Source: Front Reprod Health. 2022 Mar 3;4:781687. doi: 10.3389/frph.2022.781687 (PMC9580795; doi:10.3389/frph.2022.781687)
Supplement: Supplementary file 6 [file Table_6.docx]

| **Supplementary Table 6. Characteristics of participants at baseline according to hormonal contraceptive randomization arms.** | | | |
| --- | --- | --- | --- |
|  | **Net-En** (n=45)(34.6%) | **COC**  (n=40)(30.8%) | **CCVR**  (n=45)(34.6%) |
| **Age at screening, median yrs (IQR)** | 17 (16-18) | 17 (16-18) | 17 (16-18) |
| **BMI (kg/m^2^), median (IQR)** | 25.0 (22.7-29.7) | 25.5 (21.5-28.1) | 24.9 (21.9-27.7) |
| **STI prevalence**  Any STI(s)  Ct  Ng  Tv  Mg | 21 (46.7%)  17 (37.8%)  5 (11.1%)  4 (8.89%)  2 (4.44%) | 15 (37.5%)  12 (30.0%)  3 (7.50%)  4 (10.0%)  1 (2.50%) | 19 (42.2%)  14 (31.1%)  5 (11.1%)  4 (8.89%)  0 (0.00%) |
| **BV prevalence**  BV positive  BV intermediate  BV negative | 22 (48.8%)  5 (11.1%)  18 (40.0%) | 16 (40.0%)  4 (10.0%)  20 (50.0%) | 19 (42.2%)  3 (6.67%)  23 (51.1%) |
| **CST distribution**^1^  CST-I  CST-III  CST-IV | 8 (19.0%)  11 (26.2%)  23 (54.8%) | 9 (22.5%)  13 (32.5%)  17 (45.0%) | 11 (25.0%)  10 (22.7%)  24 (52.3%) |
| **Vaginal pH, mean (sd)**  >4.7, n (%) | 5.0 (4.3-5.6)  25 (55.6%) | 4.8 (4.3-5.4)  19 (47.5%) | 4.8 (4.3-5.4)  21 (46.7%) |
| **Shannon Index, median (IQR)**^1^ | 1.78 (0.64- 2.27) | 0.87 (0.43-1.99) | 1.46 (0.61-2.14) |
| **HSV-2 serology**^2^ | 13 (28.9%) | 14 (35.0%) | 12 (26.7%) |
| **Yeast cells present** | 4 (8.89%) | 6 (15.0%) | 10 (22.2%) |
| **Inflammation category**^3^  High  Low | 23 (56.1%)  18 (43.9%) | 20 (51.3%)  19 (48.7%) | 28 (62.2%)  17 (37.8%) |
| **Days since last menstrual period, median (IQR)**^4^ | 42 (19-105) | 41 (20-229) | 37 (14-123) |
| **Antibiotic use (past 3 months)** | 4 (8.9%) | 0 (0.00%) | 4 (8.89%) |
| **Age menarche, median (IQR)**^5^ | 13 (12-14) | 13 (12-14) | 13 (12-14) |
| **Tanner, median (IQR)**^6^ | 4.0 (4.0-4.0) | 4.0 (4.0-4.0) | 4 .0 (4.0-4.0) |
| **Parity**^9^  Previously pregnant | 7 (15.6%) | 4 (10.3%) | 6 (13.3%) |
| **Use of hormonal contraception**^7^  Naive  Not currently  Net-En  COC  DMPA  Implanon | 2 (4.44%)  10 (22.2%)  21 (46.7%)  2 (4.44%)  7 (15.6%)  2 (4.44%) | 1 (2.5%)  10 (25.0%)  20 (50.0%)  1 (2.5%)  7 (17.5%)  0 (0.0%) | 2 (4.44%)  6 (13.3%)  28 (62.2%)  3 (6.67%)  5 (11.1%)  1 (2.22%) |
| **Intra-vaginal practices**^8^  Douching  Washing with water  Washing with soap  Tampon use  Put anything else inside vagina (medication/herbs) | 1 (2.2%)  6 (13.3%)  6 (13.3%)  5 (11.1%)  4 (8.89%) | 0 (0.0%)  5 (12.8%)  2 (5.13%)  1 (2.56%)  1 (2.56%) | 0 (0.0%)  5 (11.1%)  4 (8.89%)  2 (4.65%)  2 (4.65%) |
| **Sexual risk behaviour**^9^  Age of sexual debut, median (IQR)  Any sexual partner(s) past year, n (%)  Multiple sexual partners past year, n (%)  New partner past year, n (%)  General condom use  *Never*  *Almost never*  *Not sure*  *Almost always*  *Always*  Condom use during last PV intercourse  Sex with older partner (≥5 years)  *No*  *Unsure*  *Yes*  Transactional sex  Penile-anal intercourse | 15 (14-16)  40 (93.0%)  5 (11.6%)  15 (34.9%)  6 (14.0%)  3 (6.98%)  7 (16.3%)  13 (30.2%)  14 (32.6%)  26 (60.5%)  15 (34.9%)  18 (41.9%)  10 (23.3%)  1 (2.33%)  0 (0.00%) | 15 (14-16)  37 (92.5%)  3 (7.50%)  9 (22.5%)  3 (7.50%)  4 (10.5%)  3 (7.50%)  19 (47.5%)  11 (27.5%)  25 (62.5%)  19 (47.5%)  13 (32.5%)  8 (20.0%)  0 (0.00%)  0 (0.00%) | 15 (14-16)  42 (93.3%)  4 (8.89%)  12 (26.7%)  3 (6.67%)  6 (13.3%)  5 (11.1%)  14 (31.1%)  17 (37.8%)  27 (60.0%)  16 (35.6%)  21 (46.7%)  8 (17.8%)  0 (0.00%)  4 (8.89%) |
| **Education**^10^  School attendance  Highest grade, median (IQR)  Tertiary attendance | 39 (86.7%)  10 (9-11)  0 (0.0%) | 36 (90.0%)  10 (8-11)  2 (5.00%) | 37 (84.1%)  9 (8-10)  2 (4.55%) |
| *BMI, body mass index; BV; bacterial vaginosis; CCVR*, *combined contraceptive vaginal ring*; *COC*, *combined oral contraceptives*; *CST, community state type*; *Ct,* Chlamydia trachomatis*; HSV-2, herpes simplex virus type-2 seropositive; IQR, interquartile range; LH, luteinizing hormone; Mg,* Mycoplasma genitalium*; Ng,* Neisseria gonorrhoea*; PV, penile-vaginal; sd, standard deviation; STI, sexually transmitted infection; Tv,* Trichomonas vaginalis; *yrs, years.*  *1. Based on samples with available microbiome data (COC, n=39; Net-En, n=42; CCVR, n=45)*  *2. One equivocal result (CCVR, n=1).*  *3. Based on samples with available microbiome and cytokine data (COC, n=39; Net-En, n=41; CCVR, n=45)*  *4. Missing data from two adolescents (COC, n=2; Net-En, n=0; CCVR, n=0).*  *5. Missing data from five adolescents (COC, n=4; Net-En, n=1; CCVR, n=0).*  *6. Missing data from one adolescent (COC, n=0; Net-En, n=1; CCVR, n=0).*  *7. Missing data from three adolescents (COC, n=0; Net-En, n=2; CCVR, n=1).* | | | |
