# Supplementary material for: Systems Analysis Reveals Contraceptive-Induced Alteration of Cervicovaginal Gene Expression in a Randomized Trial
Source: Front Reprod Health. 2022 Mar 3;4:781687. doi: 10.3389/frph.2022.781687 (PMC9580795; doi:10.3389/frph.2022.781687)
Supplement: Supplementary file 7 [file Table_7.docx]

| **Supplementary Table 7. Characteristics of participants with transcriptomics data at the 16-week follow-up visit stratified by study arm** | | | | |
| --- | --- | --- | --- | --- |
|  | **Net-En**  n=33 (34.4%) | **COC**  n=32 (33.3%) | **CCVR**  n=31 (32.3%) | **P value** |
| **BV prevalence**  BV positive (Nugent 7-10)  BV intermediate (Nugent 4-6)  BV negative (Nugent 0-3) | 15 (45.5%)  1 (3.1%)  17 (51.5%) | 11 (34.4%)  2 (6.3%)  19 (59.4%) | 13 (41.9%)  1 (3.3%)  17 (54.8%) | 0.869 |
| **Community state type (CST)**^1^  *CST-I*  *CST-III*  *CST-IV* | 13 (41.9%)  3 (9.7%)  15 (48.4%) | 6 (19.4%)  14 (45.2%)  11 (35.5%) | 5 (16.1%)  11 (35.5%)  15 (48.4%) | **0.014** |
| **STI prevalence**  Any bacterial/protozoan STI(s)  *Ct*  *Ng*  *Tv*  *Mg* | 8 (24.2%)  3 (9.1%)  1 (3.0%)  1 (3.0%)  3 (9.1%) | 7 (21.9%)  4 (12.5%)  2 (6.3%)  1 (3.1%)  1 (3.1%) | 13 (41.9%)  8 (25.8%)  7 (22.6%)  1 (3.2%)  0 (0.0%) | 0.161  0.178  **0.043**  1.000  0.320 |
| **HSV-2 serology**  Overall | 23 (69.7%) | 20 (62.5%) | 18 (58.1%) | 0.620 |
| **Yeast cells present** | 5 (15.2%) | 4 (12.5%) | 5 (16.1%) | 0.973 |
| **Antibiotic use since last visit** | 13 (39.4%) | 14 (43.8%) | 9 (29.0%) | 0.318 |
| **High inflammation group**^2^ | 14 (45.2%) | 13 (41.9%) | 19 (65.5%) | 0.144 |
| **Sexual behaviour since last visit**^3^  Current sexual partner  Multiple sexual partners  New partner  Sex acts per week, median (IQR)  Condom use  *Never*  *Less than half the time*  *Half the time*  *More than half the time*  *Always*  Condom use during last PV intercourse  *Yes*  Intergenerational sex with older partner (≥5 years)  *Yes*  Transactional sex  Penile-anal intercourse | 25 (86.2%)  1 (3.4%)  1 (3.4%)  2 (1-2)  9 (31.0%)  1 (3,4%)  12 (41.4%)  2 (6.9%)  5 (17.2%)  13 (44.8%)  3 (10.3%)  0 (0.0%)  0 (0.0%) | 27 (90.0%)  1 (3.3%)  2 (6.7%)  2 (1-2)  5 (16.7%)  6 (20.0%)  13 (43.3%)  3 (10.0%)  3 (10.0%)  20 (66.7%)  1 (3.3%)  0 (0.0%)  0 (0.0%) | 27 (93.1%)  0 (0.0%)  0 (0.0%)  1 (1-2)  6 (20.7%)  2 (6.9%)  6 (20.7%)  4 (13.8%)  11 (37.9%)  18 (62.1%)  0 (0.0%)  0 (0.0%)  0 (0.0%) | 0.763  0.765  0.638  0.393  0.076  0.203  0.217  NA  NA |
| *Chi-squared test (Fisher’s exact test when expected values < 5) for the assessment of association of frequency among groups, unpaired Mann–Whitney U test for comparison of medians and unpaired Student’s t test for comparison of means. BV; bacterial vaginosis; CCVR, combined contraceptive vaginal ring; CI, confidence interval, COC, combined oral contraceptives; Ct,* Chlamydia trachomatis*; Ng,* Neisseria gonorrhoeae*;* *Mg,* Mycoplasma genitalium; *STI, sexually transmitted infection; Tv,* Trichomonas vaginalis*.*  *1. Missing data from three adolescents (Net-En: n=2, COC: n=1).*  *2. Missing data from five adolescents (Net-En: n=2, COC: n=1, CCVR: n=2)*  *3. Missing data from eight adolescents (Net-En: n=4, COC: n=2, CCVR: n=2).* | | | | |
