# Supplementary material for: Systems Analysis Reveals Contraceptive-Induced Alteration of Cervicovaginal Gene Expression in a Randomized Trial
Source: Front Reprod Health. 2022 Mar 3;4:781687. doi: 10.3389/frph.2022.781687 (PMC9580795; doi:10.3389/frph.2022.781687)
Supplement: Supplementary file 12 [file Data_Sheet_1.PDF]

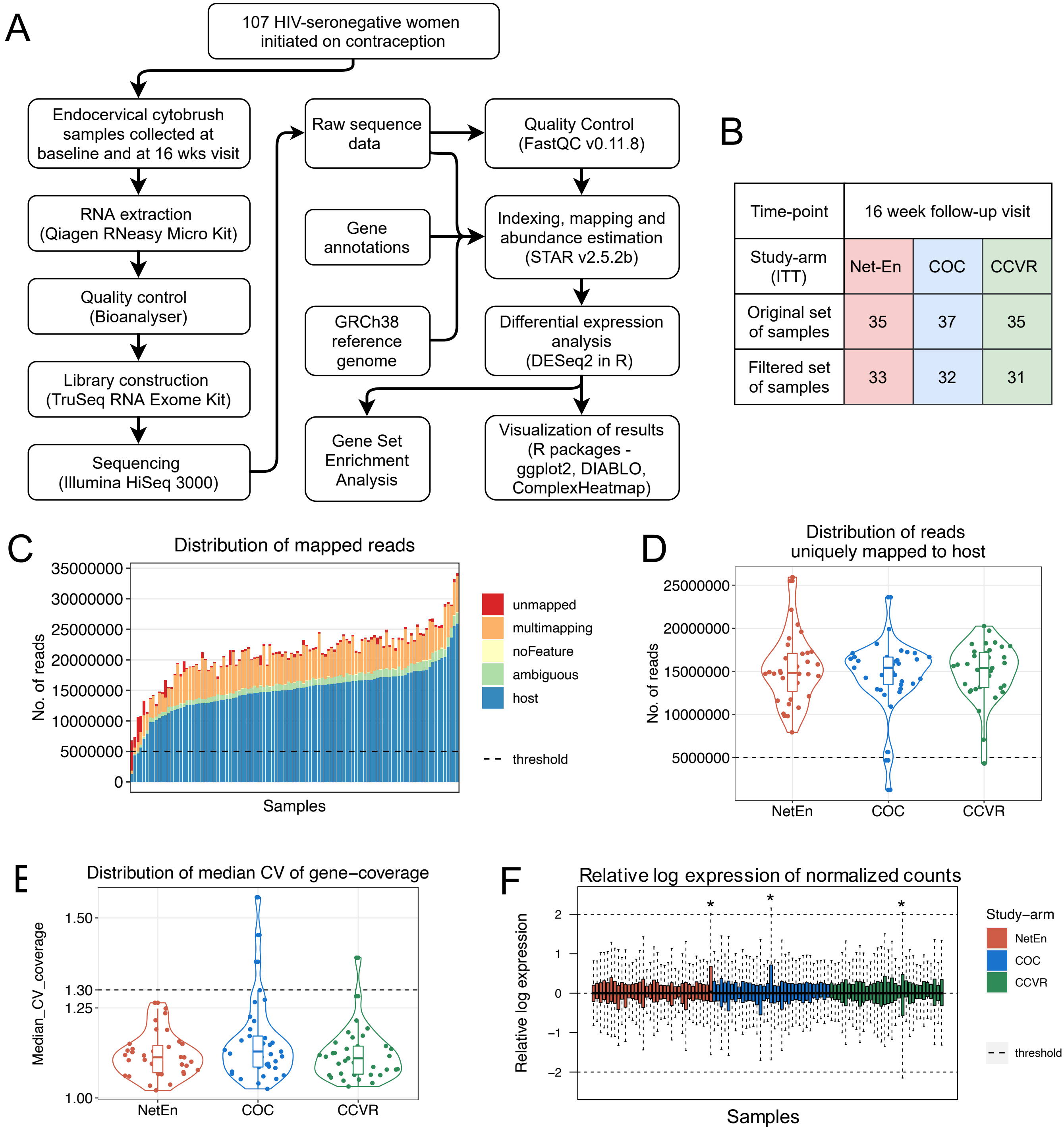

Time-point	16 week follow-up visit		
Study-arm (ITT)	Net-En	COC	CCVR
Original set of samples	35	37	35
Filtered set of samples	33	32	31

Supplementary Figure 1: A schematic representation of the RNA-Seq analysis and identification of low-quality samples. (A) The flowchart of RNA-Seq analysis outlines the experimental and data analysis steps carried out in the study. **(B)** The number of samples at collection timepoint and after quality-based filtering have been shown in the colored boxes. **(C-F)** Plots to identify selection thresholds for quality-based filtering of samples. The distributions of mapping of reads **(C)**, the number of reads uniquely mapped to the host **(D)**, the median coefficient of variation (CV) of gene coverage **(E)** and relative log expression plot of normalized counts **(F)** help to identify samples that are outliers with metrics lying in the extreme value ranges. COC, combined oral contraceptives; CCVR, combined contraceptive vaginal ring.
