# Supplementary material for: Systems Analysis Reveals Contraceptive-Induced Alteration of Cervicovaginal Gene Expression in a Randomized Trial
Source: Front Reprod Health. 2022 Mar 3;4:781687. doi: 10.3389/frph.2022.781687 (PMC9580795; doi:10.3389/frph.2022.781687)
Supplement: Supplementary file 13 [file Data_Sheet_2.PDF]

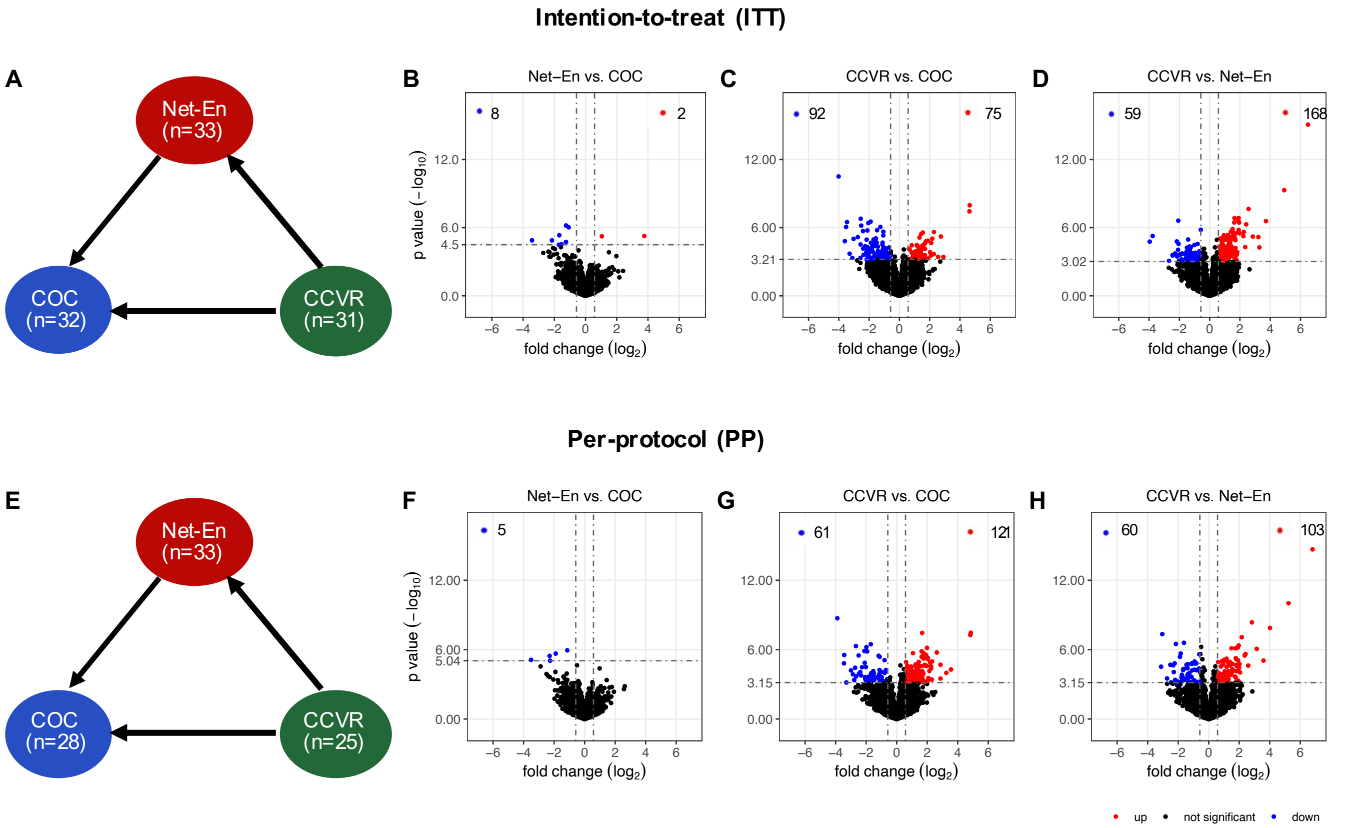


**Supplementary Figure 2. Statistical analyses on differential expression of cross-arm comparisons based on intention-to-treat (ITT) and per-protocol (PP) designs**. The significantly differentially expressed genes (DEGs) were identified by the following criteria: false discovery rate (FDR) <0.05, absolute fold-change >1.5 and standard-error in fold-change (lfcSE) <1. (**A,E**) Number of participants included in each study-arm in ITT and PP analyses is shown. Although the number of participants assigned to Net-En is the same in ITT and PP, the number is slightly lower for COC and CCVR arms in PP as compared to that in ITT. (**B-D, F-H**) Volcano plots showing statistical significance against fold-change for each comparison, with the DEGs highlighted in red (upregulated) and blue (downregulated) colors indicated in the upper quadrants.
