# Supplementary material for: Systems Analysis Reveals Contraceptive-Induced Alteration of Cervicovaginal Gene Expression in a Randomized Trial
Source: Front Reprod Health. 2022 Mar 3;4:781687. doi: 10.3389/frph.2022.781687 (PMC9580795; doi:10.3389/frph.2022.781687)
Supplement: Supplementary file 15 [file Data_Sheet_4.PDF]

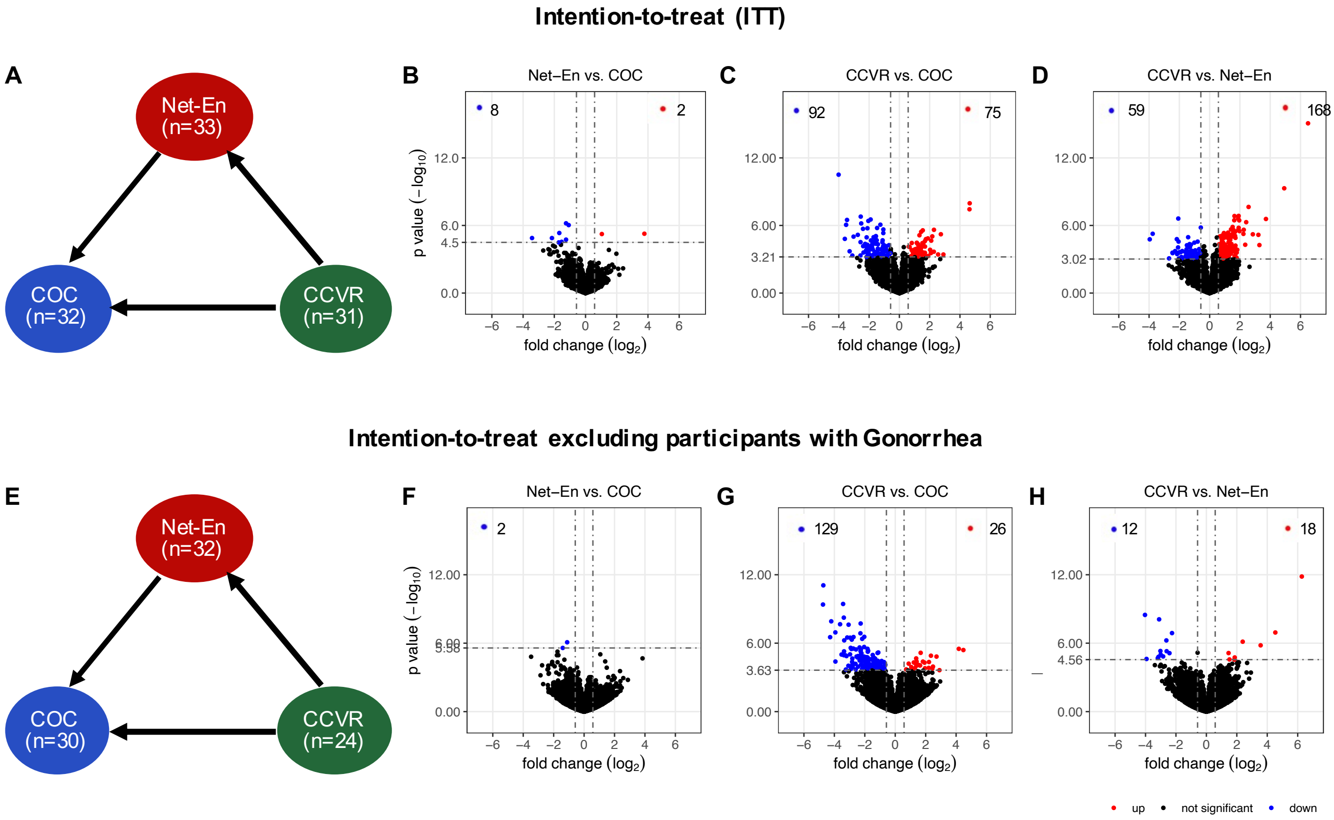

Supplementary Figure 4. Statistical analyses on differential expression of cross-arm comparisons based on intention-to-treat (ITT) including all participants and ITT excluding participants with Gonorrhea (ITT Ng-ve). The significantly differentially expressed genes (DEGs) were identified by the following criteria: false discovery rate (FDR) <0.05, absolute fold-change >1.5 and standard-error in fold-change (lfcSE) <1. (**A,E**) Number of participants included in each study-arm in ITT and ITT_Ng-ve analyses is shown. (**B-D, F-H**) Volcano plots showing statistical significance against fold-change for each comparison, with the DEGs highlighted in red (upregulated) and blue (downregulated) colors indicated in the upper quadrants.
