# Supplementary material for: Systems Analysis Reveals Contraceptive-Induced Alteration of Cervicovaginal Gene Expression in a Randomized Trial
Source: Front Reprod Health. 2022 Mar 3;4:781687. doi: 10.3389/frph.2022.781687 (PMC9580795; doi:10.3389/frph.2022.781687)
Supplement: Supplementary file 16 [file Data_Sheet_5.PDF]

**Marginal power-related results.**							
**SS1**	**SS2**	**Nominal FDR**	**Actual FDR**	**Marginal power**	**Avg # of TD**	**Avg # of FD**	**FDC**
5	5	0,05	0,22	0,6	339,7	97,4	0,287
10	10	0,05	0,134	0,76	430,45	68,35	0,159
20	20	0,05	0,094	0,873	494,3	54,15	0,11
30	30	0,05	0,075	0,913	516,7	45,05	0,087
40	40	0,05	0,061	0,938	530,95	37,85	0,071
50	50	0,05	0,059	0,954	539,65	37,4	0,069
Marginal power-related results have been shown for each pair of sample sizes (SS1 and SS2), including marginal power, true discovery (TD), false discovery (FD), and false discovery cost (FDC, defined as the number of FD divided by the number of TD).							

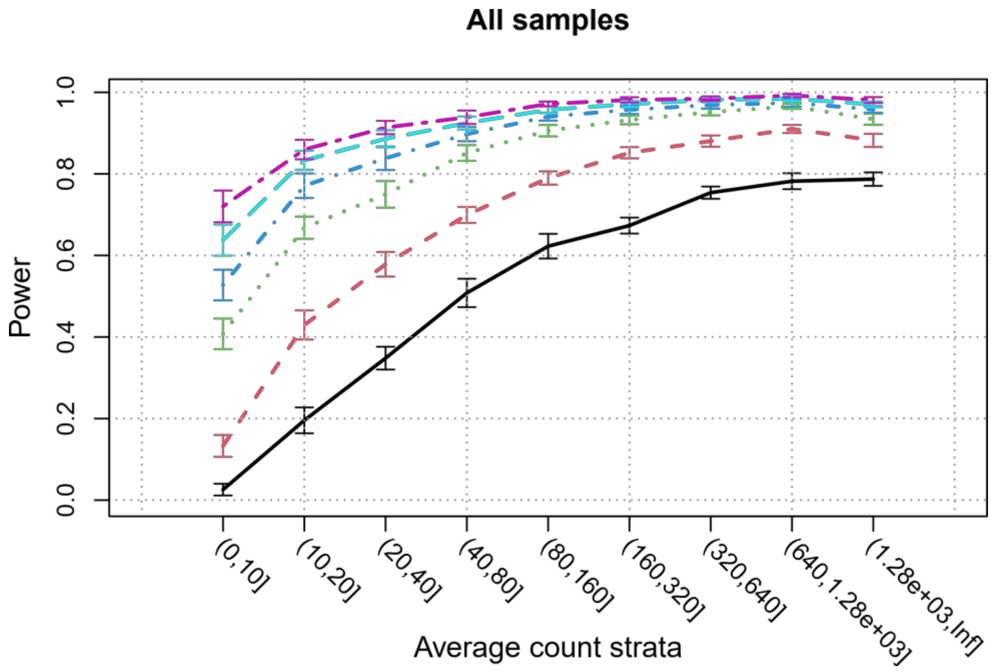

Supplementary Figure 5. The stratified power is represented by each line for a certain sample size, stratified by the average counts of genes. For a sample size of 30, the power is between 0.6 and 0.8 for genes with low counts (between 0 and 10) but improves significantly for genes with counts higher than 10 reads.
