# Supplementary material for: Systems Analysis Reveals Contraceptive-Induced Alteration of Cervicovaginal Gene Expression in a Randomized Trial
Source: Front Reprod Health. 2022 Mar 3;4:781687. doi: 10.3389/frph.2022.781687 (PMC9580795; doi:10.3389/frph.2022.781687)
Supplement: Supplementary file 17 [file Data_Sheet_6.PDF]

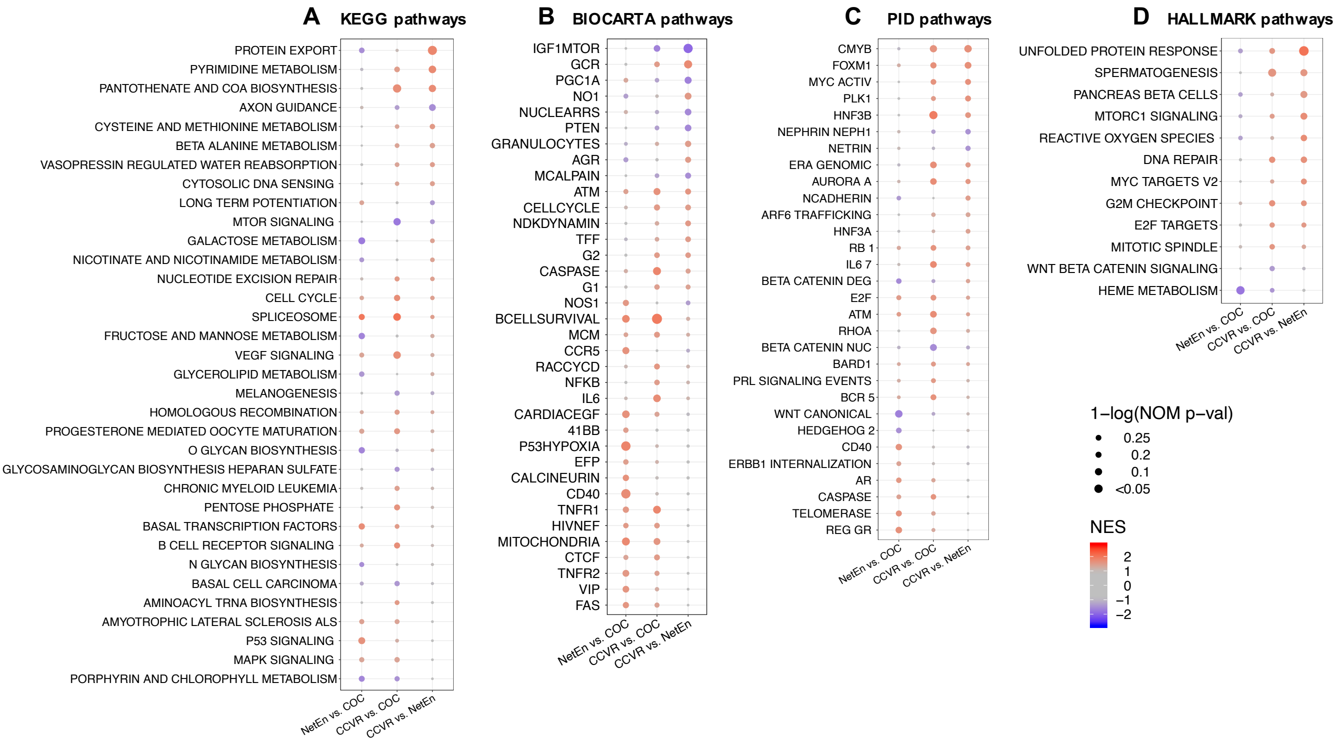

Supplementary Figure 6: Intention-to-treat: Pathways enriched in at least one of the three study arms (Net-En, combined oral contraceptives (COC) or combined contraceptive vaginal ring (CCVR)) after 16 weeks of randomized contraceptive in cross-arm comparisons (nominal p-value <0.1). Dot plots to represent the statistical significance (nominal p-values) and normalized enrichment scores (NES) in gene set enrichment analysis (GSEA), for several MSigDB pathway collections have been shown. The statistical significance of the enriched pathways is shown by the size of the dots (1-ln(nominal p-value)), the larger the dot, the higher is the statistical significance. The normalized enrichment score (NES) is represented by a blue-to-red color-gradient, blue for negative scores and red for positive scores. The pathways are ordered by the nominal p-value of CCVR vs. Net-En contrast, with the most statistically significant pathway shown at the top.
