# Supplementary material for: Systems Analysis Reveals Contraceptive-Induced Alteration of Cervicovaginal Gene Expression in a Randomized Trial
Source: Front Reprod Health. 2022 Mar 3;4:781687. doi: 10.3389/frph.2022.781687 (PMC9580795; doi:10.3389/frph.2022.781687)
Supplement: Supplementary file 18 [file Data_Sheet_7.PDF]

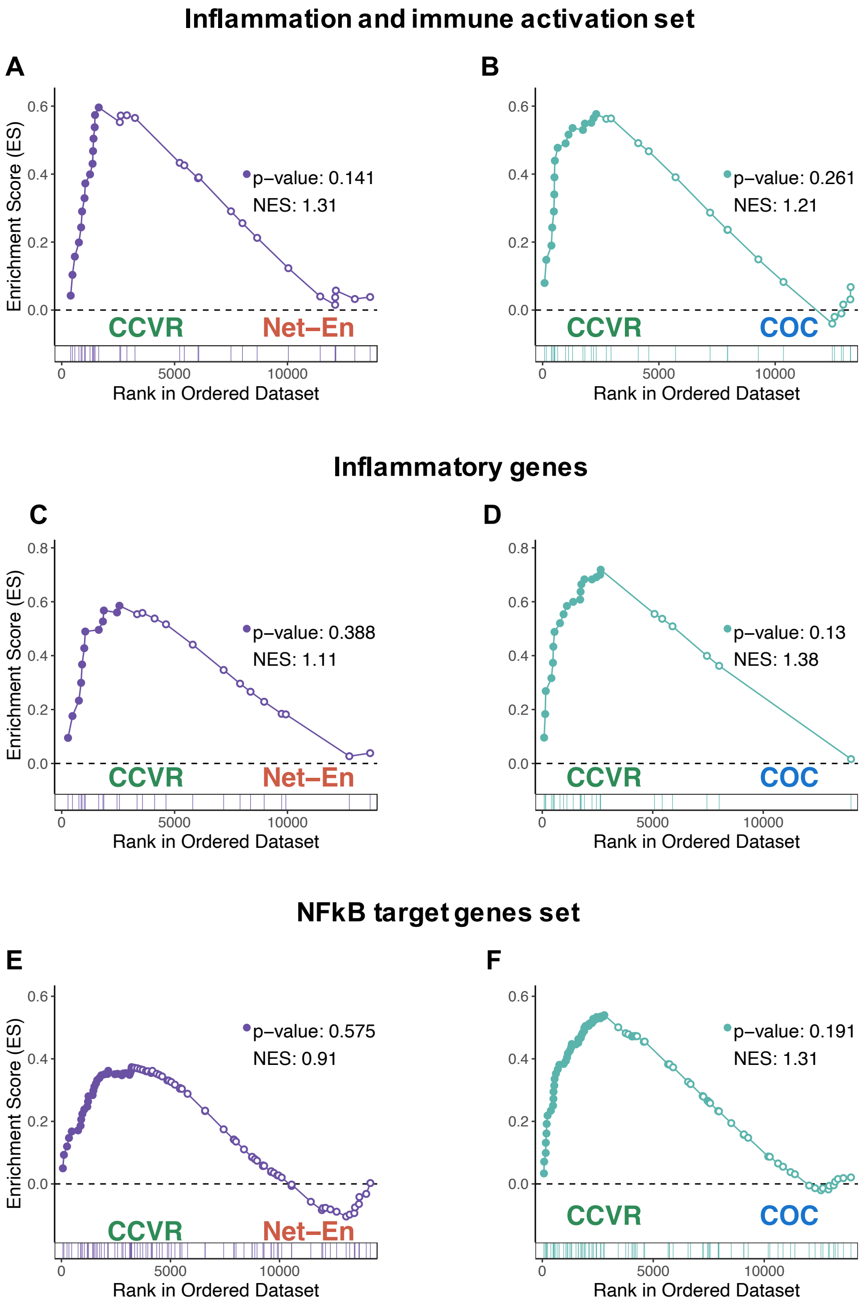

Supplementary Figure 7: Enrichment of custom gene-sets in CCVR with respect to either Net-En or COC. In the enrichment line plots, the running enrichment score (y-axis) is indicated for each gene ordered by their rank in the whole data set for that specific comparison, shown by the vertical bars shown below the x-axis. Enrichment statistics, nominal p-value and normalized enrichment scores (NES) are shown alongside each line plot for each of the cross-arm comparisons. COC, combined oral contraceptives; CCVR, combined contraceptive vaginal ring.
