# Supplementary material for: Systems Analysis Reveals Contraceptive-Induced Alteration of Cervicovaginal Gene Expression in a Randomized Trial
Source: Front Reprod Health. 2022 Mar 3;4:781687. doi: 10.3389/frph.2022.781687 (PMC9580795; doi:10.3389/frph.2022.781687)
Supplement: Supplementary file 20 [file Data_Sheet_9.PDF]

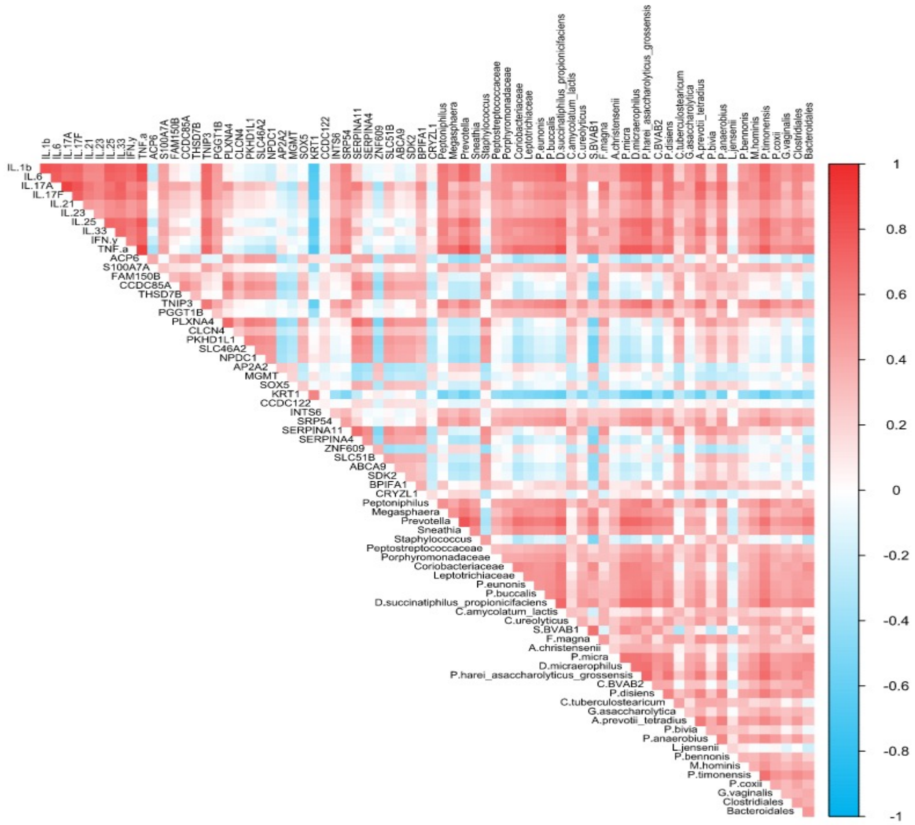

Supplementary Figure 9. Integration of gene expression, microbiome and cytokine data. (A) Circos plot depicting correlated bacteria (orange), cytokines (green) and genes (purple) identified using DIABLO analysis as most discriminatory between the study arms. The expression level of each variable is indicated by the lines outside the circle and colored according to each study arm (Net-En: red, combined oral contraceptives (COC): blue; combined contraceptive vaginal ring (CCVR): green). Positive (red) or negative (blue) correlations between individual variables are indicated with lines connecting these within the circle. Only R^2 values>0.3 displayed. **(B)** Correlation plot depicting positive (red) or negative (blue) correlation between bacteria, cytokines and genes identified using DIABLO analysis as most discriminatory between the study arms. **(C)** Significant positive (blue) or negative (red) correlations between normalized read counts of top 1000 genes and vaginal concentrations of cytokines using Spearman's rank correlation and adjusted for multiple comparisons using the Benjamini-Hochberg method. Adjusted p value of 0.01 set as cut-off. Only R^2 values>0.3 displayed.
